# Topological constraints strongly affect chromatin reconstitution *in silico*

**DOI:** 10.1093/nar/gku1085

**Published:** 2014-11-28

**Authors:** C.A. Brackley, J. Allan, D. Keszenman-Pereyra, D. Marenduzzo

**Affiliations:** 1 SUPA, School of Physics & Astronomy, University of Edinburgh, Edinburgh, EH9 3FD, UK; 2 Institute of Cell Biology, University of Edinburgh, Edinburgh, EH9 3BF, UK; 3 Edinburgh Genomics, Ashworth Laboratories, University of Edinburgh, Edinburgh, EH9 3FL, UK

## Abstract

The fundamental building block of chromatin, and of chromosomes, is the nucleosome, a composite material made up from DNA wrapped around a histone octamer. In this study we provide the first computer simulations of chromatin self-assembly, starting from DNA and histone proteins, and use these to understand the constraints which are imposed by the topology of DNA molecules on the creation of a polynucleosome chain. We take inspiration from the *in vitro* chromatin reconstitution protocols which are used in many experimental studies. Our simulations indicate that during self-assembly, nucleosomes can fall into a number of topological traps (or local folding defects), and this may eventually lead to the formation of disordered structures, characterised by nucleosome clustering. Remarkably though, by introducing the action of topological enzymes such as type I and II topoisomerase, most of these defects can be avoided and the result is an ordered 10-nm chromatin fibre. These findings provide new insight into the biophysics of chromatin formation, both in the context of reconstitution *in vitro* and in terms of the topological constraints which must be overcome during *de novo* nucleosome formation *in vivo*, e.g. following DNA replication or repair.

## INTRODUCTION

Chromatin is the functional form of DNA within the nuclei of eukaryotic cells, and it is the building block of chromosomes. It is a composite material made up, in its simplest form, of DNA wrapped around positively charged histone octamers (1). Chromatin provides a first level of DNA compaction, and at the same time gives more mechanical strength to the genetic material, which is necessary for instance during mitosis as microtubules pull the chromosomes apart (2). Electron microscopy studies of the chromatin fibre at low salt reveal a ‘beads-on-a-string’ structure, also known as the 10-nm fibre, where individual nucleosomes—histone octamers with ∼147 base pairs of DNA wrapped around them—are separated from each other (1,3). *In vivo*, additional interactions, mediated by histone H1 and other proteins, lead to further compaction, either into the so-called 30-nm fibre (2), or into more complex and less-ordered structures (4,5). Despite this hierarchical packaging, the DNA must also remain accessible since, for example, transcription requires the local displacement of histones from the DNA to allow other proteins, such as polymerases, to bind (6). Similarly, during replication nucleosomes must be displaced as the replication fork passes, with new nucleosomes assembled on the newly replicated DNA.

In this work we use computer simulations to help us understand nucleosome formation on chromatin fibres. *In vivo* this is a complex process involving a variety of chromatin assembly proteins, which act as energy consuming molecular machines that position and assemble nucleosomes. However, it is also possible for nucleosomes to form without this complicated machinery, and indeed this occurs regularly in the lab. Our aim here is not to simulate the detailed process of *in vivo* nucleosome formation, but rather to investigate the pathway by which chromatin fibres can self-assemble from their constituent parts, for instance *in vitro*, focussing particularly on the role of DNA topology and supercoiling. In order to wrap around a histone octamer, DNA needs to acquire two negative units of writhe: this introduces locally a twist excess and/or supercoils, which one imagines will need to diffuse away for appropriate chromatin self-assembly to progress. In other words, nucleosome formation potentially brings about topological issues which, if left untreated, can hinder reconstitution of a 10-nm fibre and create defects. In our simulations we confirm the importance of these considerations, even for only moderately long, linear DNA molecules, and we identify the nature of the chromatin defects associated to these topological constraints, as well as possible mechanisms by which they can be avoided. Besides helping us to understand chromatin reconstitution *in vitro*, these simulations also give insight into the obstacles which must be overcome for *in vivo* nucleosome formation, by the cellular chromatin assembly machinery.

Our approach is to use coarse grained modelling, a strategy which has had success in studying *pre-assembled* fibres—see, for example, the seminal works in (7–14). These previous models have treated nucleosomes as rigid objects (where the DNA cannot unwrap) connected by linker DNA, and there has been to date no treatment of chromatin reconstitution *in silico*; our goal here is to fill this gap and investigate the self-assembly of a 10-nm chromatin fibre from DNA and histone proteins. Since we do not address the formation of higher order chromatin structures beyond the 10-nm fibre, we disregard here histone–histone interactions due to histone tails and histone surface charge distributions, which have been treated elsewhere (9,15). Our work is also quite distinct from previous investigations of the formation of a *single* nucleosome with a spherical core (16,17); as we shall see below, modelling DNA-mediated interactions between different nucleosomes is absolutely essential if we are to understand assembly of entire 10-nm fibres.

As mentioned above, many recent experiments investigating the properties of chromatin rely on the use of artificially created fibres, rather than those harvested from living cells (18–24). Such ‘reconstituted’ chromatin is usually formed via one of two methods: either by salt gradient dialysis (18–21) from purified histones and DNA or by assembly using the same constituents supplemented with a cell extract (e.g. from Xenopus oocytes) (22–24). Since it is performed in the absence of cellular machinery, we draw inspiration from the salt gradient dialysis method to inform our model.

Attempts to reconstitute chromatin *in vitro* by simply mixing DNA and purified core histones at a physiological salt concentration of 100 mM NaCl are not particularly successful, often resulting in the production of disordered aggregates and extensive precipitation (3,6). Furthermore, experiments studying the self-assembly of ‘synthetic chromatin’ from bacteriophage DNA and charged nanoparticles also lead to the formation of nanoparticle aggregates on the DNA (25,26) (even though the wrapping of short DNA around a single nanoparticle does resemble that observed for real nucleosomes). To obtain realistic chromatin fibres *in vitro* a precise reconstitution protocol must be followed, and our work helps to explain why this is so, since we can control the position of all constituents at any time.

## MATERIALS AND METHODS

We perform coarse grained Brownian dynamics simulations using the lammps (Large-scale Atomic/Molecular Massively Parallel Simulator) software package (27). Proteins are modelled as groups of spheres which move and rotate as rigid bodies; DNA molecules are modelled as ‘bead-and-spring’ polymers, where each bead moves as a rigid body, and consists of a core and a ‘patch’. The latter acts as a protein-binding site, and is used to give the polymer torsional rigidity (i.e. it can support supercoiling). DNA thickness, persistence length and torsional rigidity were set to 2.5 nm, 50 nm and 3× 10^−19^ erg cm respectively—all in line with known properties of DNA in a physiological salt buffer (28,29). lammps was run in Brownian dynamics mode, where a molecular dynamics algorithm is used with a stochastic thermostat, which models the thermal fluctuations and viscosity of an implicit solvent. For computational efficiency hydrodynamic interactions are neglected; we note that this approximations will change the way in which the polymer relaxation time scales with its length (qualitatively we do not expect these issues to affect our findings, see Supplementary Data). This scheme is often referred to as Langevin dynamics, and is a common method used in many recent DNA and chromatin simulations (30–32). The solvent viscosity is chosen so as to reproduce the Brownian time (the characteristic time it takes for a protein or DNA bead to diffuse across its own diameter) known through the Stokes–Einstein relation for an object diffusing in water. Full details of the mapping between simulation and real times are given in the Supplementary Data. To describe the interactions between the different components we use phenomenological force fields. This is much more computationally efficient than a full description of the charges in the system, and since for the 100 mM (and higher) salt concentrations considered the Debye screening length is of the order 1 nm (and shorter), this is unlikely to affect our results. Again, full details are given in the Supplementary Data.

## RESULTS

### Isotropic DNA–histone interaction leads to aggregation

**Figure 1.**
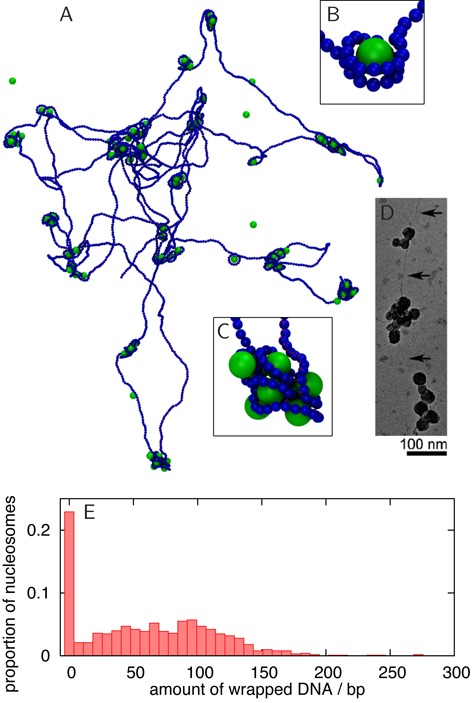
Spherical nanoparticle model synthetic nucleosomes. (A) Snapshot from an MD simulation of a 22.1 kbp DNA molecule interacting with 100 spherical nanoparticles (diameter 6.75 nm), taken 0.18 ms after the system was initialised with nanoparticles positioned randomly and the DNA in an equilibrium configuration. The spheres and DNA interact with an energy 4.3*k*_*B*_*T*. The *in silico* reconstitution leads to the formation of nanoparticle–DNA aggregates. (B) Snapshot of a nanoparticle wrapped by two turns of DNA, as in chromatin: this structure is very rare in the simulation. (C) Detailed view of a nanoparticle–DNA cluster. (D) Transmission electron microscopy image of T4 DNA in complex with poly(*L*-lysine)-coated silica nanoparticles of diameter 15.1 nm (see (25) for details). Clusters of nanoparticles similar to those in simulations are observed. Reproduced from Figure 2f in (25) with permission. ‘Copyright (2005) by the American Physical Society.’ (E) Histogram from simulation data showing how much DNA is associated with each particle, including data from 10 different simulations. A canonical nucleosome will wrap ∼147 bp of DNA in ∼1.75 turns. Clearly, most spheres are associated with an ‘incorrect’ amount of DNA, and indeed the self-assembled structure is very different from a normal chromatin fibre.

As noted above, nucleosome cores have previously (16,26) been modelled as simple spheres which interact isotropically with DNA—this is a fairly realistic representation of charged nanoparticles, as have been used *in vitro* as ‘synthetic’ nucleosome cores (25). We consider this case here as well, as the simplest model which illustrates some of the generic problems that the self-assembly process needs to overcome. Figure 1 shows the result from a computer simulation recreating the self-assembly of the ‘synthetic chromatin’ of (25) from a mixture of DNA and nanoparticles. The simulation confirms the formation of large nanoparticle aggregates as observed in the experiment (25,26) (compare, for instance, Figure 1A with the transmission electron micrograph in Figure 1D).

Our simulation (see Supplementary Data for details and a full list of parameter values) started with a dilute solution of spherical nanoparticles interacting with a single DNA molecule (Figure 1B and Supplementary Movie 1). There is an attraction between the nanoparticles and the beads making up the DNA backbone which is strong enough that an isolated sphere will wrap two turns of DNA (Figure 1C, Supplementary Movie 1), as appropriate both for real chromatin and the synthetic analogue considered in (25) (the precise affinity value chosen does not substantially change our results, see Supplementary Figure S2A).

The formation of nanoparticle clusters (Figure 1A, C, D, and Supplementary Figure S2) is an example of an aggregation phenomenon which occurs in general for proteins which can bind non-specifically to the genome at multiple points (e.g. forming bridges (33)). The clustering is due, in this case, mainly to the fact that binding of a nanoparticle induces a local increase in DNA density, which recruits further nanoparticles, triggering a positive feedback loop. Similar effects are observed to some extent in other systems, such as polyelectrolytes in ionic environments (34).

**Figure 2.**
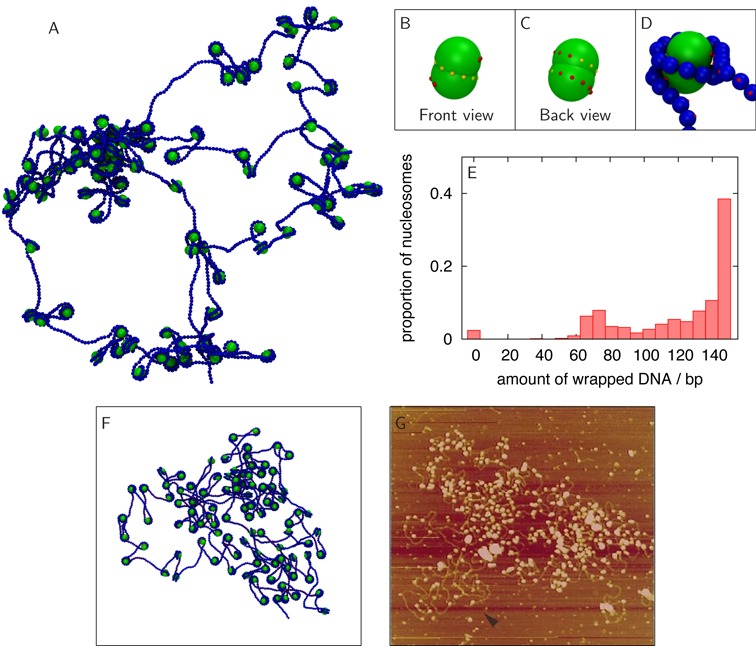
Model nucleosomes with a helical path of binding sites switched on hierarchically form a more realistic chromatin fibre, but with some defects. (A) Snapshot showing a simulation of 100 nucleosome cores and a 22.1 kbp DNA molecule, after 0.3 ms. The image is a projection of a 3D configuration. Initial conditions were to position the cores at regular intervals along a DNA molecule in an equilibrium conformation. (B,C) Each model octamer has a helical binding path which can make contact with the minor groove of the model DNA (Supplementary Figure S1, Supplementary Movie S1). (D) A correctly wrapped nucleosome, with the DNA binding along the helical path on the model histone. (E) Histogram showing how much DNA is associated with each core (i.e. the amount of DNA wrapped), including data from 10 different simulations with identical parameters to that shown in (A). It can be seen that around 40% of the cores are in correctly formed nucleosomes (which wrap ∼147 bp of DNA). (F) Image from a simulation similar to that shown in (A), but here the structure has been flattened onto a plane for better comparison with microscopy images (see Supplementary Data and Supplementary Figure S5 for details). (G) Atomic force microscopy image of a chromatin fibre from a salt dialysis experiment performed on a long DNA loop (see (41) for details). An uneven distribution of nucleosomes is shown, and this could result from defects similar to those found in simulations. Reproduced with permission from Figure 4 in (41).

The simulation in Figure 1 arguably captures the main features of the synthetic DNA–nanoparticle chromatin studied in (25), and it is also useful because it highlights the non-specific clustering effect which may in principle hinder the self-assembly of a regular structure, such as the 10-nm chromatin fibre, from a solution of histone proteins and DNA. On the other hand, *in vitro* experiments clearly show that such regular fibres *can* be obtained *in vitro* by reconstitution with chaperones (35,36), or by salt dialysis with relatively small DNA molecules (18–21). What is, then, the origin of the shortcomings of the simple model in Figure 1? An analysis of the crystal structure of the nucleosome (37) shows that the histone proteins contact the DNA backbone at regular intervals along the minor groove, and that the binding sites on the surface of the histone octamer are arranged along a left-handed helical path which gives rise to the left-handed wrapping of DNA around the octamer. We therefore adapted our model to incorporate a histone core with a helical path of binding regions (Supplementary Movie S1, Figure 2B and C; a similar approach was used to previously simulate a *single* nucleosome (17)).

### Helically arranged DNA–histone binding sites reduce clustering, but defects still form

Results from an *in silico* reconstitution where histone octamers are modelled as particles with patches corresponding to binding regions arranged helically on the surface are shown in Figure 2 (see also Supplementary Movie S2 and Supplementary Data). The octamer binding sites interact with complementary patches on the DNA, modelling negatively charged regions on the minor groove of B-DNA (Supplementary Figure S1). As the core histones do not form a stable octamer at physiological ionic strength in the absence of DNA binding, we further modified the binding interaction so as to switch on the attraction for the set of binding sites corresponding to the H3.H4 tetramer, run our reconstitution simulations for a set time, and only thereafter switch on the remaining binding sites corresponding to the H2A.H2B dimers (see Supplementary Data for details). This scenario is analogous to the kinetic pathway followed during salt dialysis, where the histone H3.H4 tetramer binds to the DNA first and subsequently, at lower salt, two H2A.H2B dimers bind to complete the octamer (18–20); this assembly pathway may also be relevant *in vivo* (38). In the simulation shown in Figure 2 we also started with histone octamers placed at regular intervals along the DNA molecule (see Supplementary Data). This might correspond in practice, for example, to using a DNA molecule with a sequence leading to strong peaks in the DNA–histone affinity (i.e. containing strong nucleosome positioning sequences such as 601 arrays (18,39)). The resulting self-assembled structure (shown in Figure 2A) is less disordered than the aggregates in Figure 1, but still far from an ordered chromatin fibre. To quantify the extent of ‘successful’ DNA–histone complex formation, we computed the distribution of the length of DNA associated with each histone octamer (Figure 2E). For perfect self-assembly we would expect each of the octamers to be associated with about 147 bp of DNA, and any deviation from this value signals the presence of defects. As can be seen from the histogram (Figure 2E), even with a helical binding path on regularly spaced octamers and a two-step reconstitution protocol, less than 50% of the DNA ends up properly wrapped to form typical nucleosomes. (As expected, a single-step reconstitution where all histone–DNA interactions are switched on from the start of the simulation leads to slightly poorer results, Supplementary Figure S3.) Nevertheless, the revised procedure provides a notable improvement over that achieved in Figure 1.

### The majority of defects arise due to topological constraints imposed by the DNA

**Figure 3.**
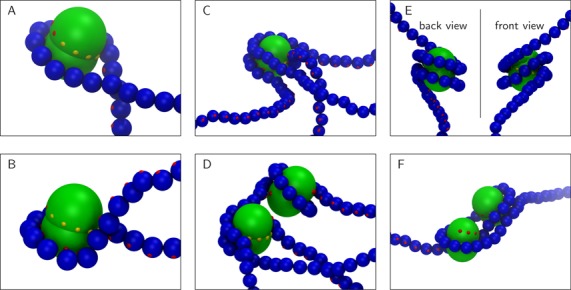
Close-up views of common self-assembly defects found in the simulation presented in Figure 2. (A,B) Partially wrapped nucleosome cores. Full wrapping in (A) would require either enough time for the strands to move around each other (i.e. time for the configuration of the entire DNA molecule to change), or strand passing. In (B) the configuration in (A) has been ‘locked’ due to a further contact between the DNA next to the already wrapped segment and one of the yellow exposed binding patches on the octamer. Resolution of the defect now requires even more time, as the non-canonical binding must first be reversed. (C,D) The exposed binding sites in partially wrapped cores can interact with DNA regions which are far away in sequence; this can lead to loop formation. In (D) this disrupts the wrapping of another nearby nucleosome. (E) DNA wrapping in a right-handed configuration around the core. The DNA does not follow the left-handed path of the core binding sites, and some binding sites remain exposed. (F) Full wrapping can be inhibited if nucleosome cores bind in close proximity along the DNA.

What exactly goes wrong during the *in silico* reconstitution in Figure 2? To address this issue, we show in Figure 3 (and Supplementary Movie 3) a set of DNA–histone conformations which correspond to the most common self-assembly defects observed in our simulations. First, histones might be only partially wrapped by DNA (Figure 3A and B); this leaves binding sites on the octamer surface that cannot be easily accessed by the appropriate, adjacent DNA without strand crossing (see Figure 3A). Partially wrapped structures are the most common defects found in our *in silico* self-assembly; these can lead to inappropriate interactions as the exposed binding sites on the histone octamer are potential binding sites for distant DNA segments, which can lead to loop formation (Figure 3C and D). Another, rarer, defect is a right-handed nucleosome (Figure 3E), as opposed to the correct left-handed helical wrapping of DNA around the octamer. A close look at Figure 3E reveals that the most obvious path from the metastable right-handed nucleosome to the left-handed one would again entail strand crossing. In general, the majority of these defects occur due to topological constraints arising due to the DNA conformation, and affecting both the interaction between DNA segments and the overall DNA dynamics. Finally, in our simulations it may happen that separate histone octamers bind close to each other along the DNA, and this inhibits complete wrapping (resulting in the dimer structures in Figure 3F). While in principle octamers can diffuse along the DNA, in practice this process is very slow (40), hence it is likely that distant DNA binds the remaining exposed binding sites leading to more complex clusters. Further details of how we classify the different kinds of defect are given in the Supplementary Data.

While our self-assembled structures are quite distinct from a regular 10-nm fibre, it is of interest to compare these configurations (Figure 2A, Supplementary Movie 2) to microscopy images of chromatin reconstituted *in vitro* by salt dialysis (41). To aid visual comparison, we have simulated deposition of the self-assembled chromatin onto a plane (see Supplementary Data for details). We see a striking similarity between the *in silico* structure in Figure 2F and the atomic force microscopy (AFM) image in Figure 2G—large clusters of nucleosomes are visible. The image (Figure 2G reproduced from (41)) shows a long (106 kbp) circular DNA molecule, onto which nucleosomes have been reconstituted via an *in vitro* salt dialysis experiment. We suggest that with such long DNA molecules, just as in our simulations in Figure 2, there is not enough time during salt dialysis to allow for the topological and conformational rearrangements which would be needed to resolve defects such as those in Figure 3.

There are other examples of defects in microscopy images which appear morphologically similar to those which we have found *in silico*. Supplementary Figure S4 shows some images of possible defects reconstituted onto different DNA templates, including large and small plasmids, and linear DNA molecules. These include loop defects (Supplementary Figure S4D), where four distinct DNA strands touch or enter the nucleosome (42), and nucleosomes with widely different angles between the entry and exit strands, which might reflect histone octamers with different amounts of DNA wrapping (Supplementary Figure S4E). Furthermore, we note that single molecule experiments show that when a nucleosomal array is twisted using magnetic tweezers (43,44), left-hand wrapped nucleosomes can convert to right-hand wrapping (similar in structure to the defect we show in Figure 3E) due to torsional stress.

### Nucleosomes store DNA twist

Since the topology of the DNA plays a major role in the self-assembly of nucleosomes, then it is essential that our simulations take account of this as accurately as possible. The ∼1.75 turns of DNA wrapped into a nucleosome constitute, in the language of supercoiling, ∼1.75 negative units of writhe. For a closed loop of DNA, the number of times the two DNA strands are wound around each other, known as the linking number (Lk), is fixed, and can be partitioned into twist (Tw) and writhe (Wr), with Lk = Tw + Wr. Experiments looking at closed DNA loops containing nucleosomes revealed that the linking-number change when the loop is topologically relaxed is only ΔLk ≈ −1.01 per nucleosome, rather than the expected ΔLk ≈ −1.75; this is known as the ‘linking number paradox’ (or the ‘nucleosome paradox’) (45). The paradox was resolved when the high-resolution crystal structure revealed that the DNA within a nucleosome is twisted and stretched, and that the helical path is not regular (46). To reproduce the same topological conditions in our simulations, we modify the DNA model such that when wrapped onto the nucleosome core, a twist of ΔTw = +0.75 is stored, so that total linking number change associated with a wrapped nucleosome is ΔLk ≈ −1, as found experimentally (see Supplementary Data for details). Unless otherwise stated, all simulations here on refer to this case.

**Figure 4.**
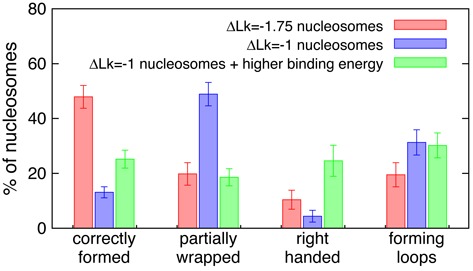
Storing DNA twist within a nucleosome does not reduce defects. Plot showing the number of occurrences of correctly wrapped nucleosomes and the various defects, for the case where twist is stored within a nucleosome (blue), and the case where it is not (red). Also shown is the case where twist is stored in the nucleosome, and the strength of the binding interaction between the DNA and nucleosome core has been increased, so as to partially offset the energy cost of twisting the DNA (green). Results show an average over 10 simulations each with 100 nucleosomes forming on a 22.1 kbp DNA molecule, where initial conditions were nucleosome cores pre-positioned at regular intervals along a DNA molecule in an equilibrium configuration.

Figure 4 shows the difference between a simulation with nucleosomes which involve a linking number change of ΔLk = −1.75, and a simulation with nucleosomes which involve a more realistic ΔLk = −1.0. Interestingly, although a smaller topological change is required on formation of ΔLk = −1.0 nucleosomes, the number of defects actually increases, and there is a statistically significant decrease in the mean of the distribution of the number of correctly formed nucleosomes across 10 simulations (*P* < 10^−5^ according to Welch's *t*-test). This is likely to be because, although the overall topological changed required for correct nucleosome formation is smaller, defects which require strand crossing for their resolution still often form. Interestingly, increasing the binding energy for wrapping (to ‘offset’ the energy cost in twisting the DNA) improves things slightly, but there are still more defects than in the case of ΔLk = −1.75 nucleosomes (see Figure 4).

### Relaxing topological constraints leads to resolution of most defects

**Figure 5.**
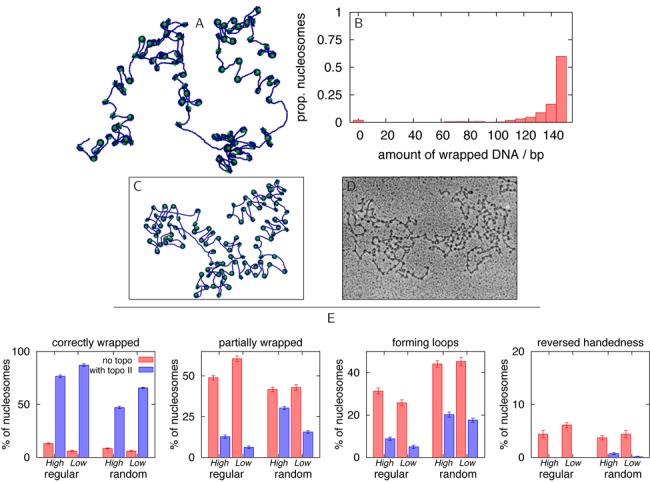
Chromatin self-assembly in the presence of topo-II like action. (A) Snapshot showing a simulation of 100 nucleosome cores and a 22.1 kbp DNA molecule, after 0.3 ms, using the ΔLk = −1.0 model nucleosomes. The image is a projection of a 3D configuration. Initial conditions where the cores are pre-positioned at regularly spaced intervals along a DNA molecule in an equilibrium conformation are used. The action of the enzyme topo-II was modelled by allowing the strands to pass though each other for about 0.1 ms, 0.1 ms after the simulation started (see Supplementary Data). (B) Histogram from simulation data showing how much DNA is associated with each core (i.e. the amount of DNA wrapped), including data from 10 different simulations with identical parameters to that shown in (A). A correctly formed nucleosome will wrap ∼147 bp of DNA in ∼1.75 turns. (C) Image from a simulation similar to that shown in (A), but here the structure has been flattened onto a plane for better comparison with microscopy images. (D) EM image of a native chromatin fibre which has been extracted from chicken erythrocytes, stripped of H1 and H5 linker histones, and treated with tripsin to remove histone tails (see (47) for details). Reproduced with permission from Figure 7c in (47). (E) Histograms from simulation data counting the numbers of correctly formed nucleosomes and defects for the reconstitutions in Figures 2 and 4, and for different initial conditions (regularly or randomly pre-positioned cores), and for high and low nucleosome density (see Supplementary Data for exact values).

Our results demonstrate that, remarkably, most of the local entanglements that give rise to defect formation are resolved if we allow strand passing during our simulation (see Supplementary Data for details). Biologically, this could correspond to the presence in the buffer of topological enzymes such as type II topoisomerase (topo-II), which use chemical energy to break and rejoin the DNA locally. Figure 5 shows the result of a self-assembly simulation, where strand crossing is allowed. The resulting reconstituted fibre is now strikingly similar to a classic 10-nm fibre (1,2,47), with the defects in Figure 3 all but absent (see Figure 5A, B and Figure 5E, Supplementary Movie 2 and Supplementary Table S1; it is also intriguing to note the zig-zagging nature of some portions of the fibre—see discussion section). In each of the cases investigated the mean of the distribution of the number of correctly formed nucleosomes across 10 simulations shows a statistically significant increase on addition of the topoisomerase-like action (*P* < 10^−5^ according to Welch's *t*-test; e.g. for the high-density regular spaced case the number of correctly formed nucleosomes increases from 13% to 77%, *t* = 42.2, *P* < 10^−12^).

**Figure 6.**
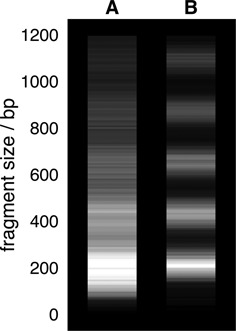
Simulated nuclease digestion experiment. *In silico* self-assembled chromatin fibre configurations were used to generate simulated gel electrophoresis analyses of fragments in a nuclease digestion experiment (see Supplementary Data for details and also Supplementary Figure S9). (A) Results from 10 simulations of 22.1 kbp DNA with 100 nucleosome cores without any topoisomerase action (i.e. using fibres such as described in Figure 2). (B) Results from 10 simulations of 22.1 kbp DNA with 100 nucleosome cores with topoisomerase II action (i.e. using fibres such as described in Figure 5). In both cases the ΔLk = −1.0 model nucleosomes were used.

To further quantify the improvement in the quality of the chromatin fibres reconstituted with topo-II, we simulated a digestion experiment, in which any DNA which is not protected by binding to a histone octamer is cut at random points by micrococcal nuclease (see Supplementary Data and (48)). The resulting digestion pattern, presented in Figure 6 as a simulated gel electrophoresis analysis, shows a characteristic ladder with bands corresponding to different numbers of nucleosomes. In stark contrast, when simulating digestion for chromatin reconstituted *in silico* without topo-II, there is a much less distinct pattern.

We would stress that our simulated digestion is designed to assess a fixed chromatin structure so as to reflect the state attained at the end of assembly. In reality, in typical experiments the structure would rearrange during nuclease digestion as partially wrapped nucleosomes could wrap more fully once the nuclease cuts and releases the nearby DNA. Such rearrangements would decrease the difference between the self-assembly with and without topo-II. However, defects other than the partially wrapped nucleosome, such as large clusters or nucleosomes forming loops, are less likely to rearrange due to a nuclease cut, as thermal nucleosome diffusion is so slow (40), hence we expect that digestion patterns would be able to identify these in reconstituted fibres. Indeed, nuclease digestion experiments assessing nucleosome footprinting have unveiled subtle differences in positively supercoiled yeast minichromosomes (49) which supported the idea that non-standard nucleosomes could form on these DNA molecules.

We explain the striking improvement in chromatin structure in Figure 5 by observing that partially wrapped histones (as in Figure 3A) lead to a juxtaposition between their entry and exiting strands that can be acted upon by topo-II, allowing the DNA to complete the correct wrapping, and hence hinder the formation of other defects (an example of this process is shown in Supplementary Movie 4; a similar mechanism was hypothesised to lead to chirality inversion in reversomes *in vivo* (50)).

Simulating type I topoisomerase (topo-I)-like activity (by allowing the DNA to be over-wound or under-wound locally at little cost) also leads to a reconstitution whose quality is close to that shown in Figure 5 (Supplementary Data and Supplementary Figures S5 and S8); the resolution of topological entanglement therefore does *not* necessarily require strand crossing. The topo-I aided conversion between a partially wrapped and a fully wrapped nucleosome is shown in Supplementary Movie 4; it requires both the flipping of the histone octamer, and the twisting of a DNA region close to the octamer, which finally snaps as if in the presence of a DNA single-strand break, allowing full wrapping. Our results are consistent with the observation that topo-I and topo-II are both able to remove supercoiling in yeast minichromosomes *in vitro* (49), although experimentally topo-II was found to do so more quickly.

As previously discussed the initial configuration we chose for the simulations in Figures 2 and 5 has all nucleosome cores pre-positioned regularly along the fibre. Starting with cores which are positioned randomly on the DNA (which corresponds to the case of a sequence with weak or no nucleosome positioning information) leads to similar results, although the quality of the reconstituted chromatin fibre is somewhat worse (Figure 5E, Supplementary Figures S6 and S8, and Supplementary Table S1). The density of nucleosomes on the DNA (i.e. the relative abundance of histones and DNA used in the reconstitution) also has an effect: as might be expected, increasing the length of the DNA for the same number of nucleosome cores (equivalently increasing the average spacing between them in the final fibre) leads to fewer instances of some types of defect, both in the case of initially regularly and randomly positioned cores (Figure 5E and Supplementary Figures S7 and S8); we observe fewer zig-zagging regions, which presumably arise due to effective interactions between nearby nucleosomes (see discussion section).

## DISCUSSION

In our simulations defects often form as nucleosomes assemble on chromatin fibres. By relaxing topological constrains imposed by the local DNA conformation and DNA–DNA interactions (such as is done by topoisomerase enzymes), most of these defects can be avoided. In other words, our simulations demonstrate that DNA topology can be an obstacle to correct nucleosome formation, even for the relatively short linear DNA molecules which we consider. Some of these defects could also be resolved naturally if given enough time (of the order of the polymer relaxation time, which is expected to scale with the square of the polymer length (51)). This explains why a typical salt dialysis reconstitution experiment must be performed over a prolonged time (∼30–36 h (52)), and can yield poor results for long DNA molecules—sufficient time must be given to allow DNA rearrangement. In our *in silico* reconstitution, there is not enough time for such rearrangements to occur, and instead the action of topoisomerases must be employed to resolve defects; the mechanism by which this occurs relies on the biophysics of supercoiling (6,53), and can be understood as follows. Wrapping twice around a nucleosome core introduces two units of writhe in the DNA; although a linear DNA molecule is not topologically closed (hence the linking number, equal to twist+writhe, is not constant), it still takes a finite time for torsional strain (supercoils) to diffuse along the molecule, especially since twist fluctuations are hindered at the histone octamer binding sites. For example in the partially wrapped nucleosome in Figure 3A, the single right-handed turn about the octamer constitutes a writhe of ∼+1, to convert this to the ∼−2 of writhe imposed by a correctly formed nucleosome, would require introducing a positive twist of ∼+3 into the DNA between the nucleosomes, and then rearranging the entire DNA so as to release the excess twist at the ends of the molecule (which takes a long time). As we have seen, the action of an enzyme which can locally change the linking number leads to much faster progress. Topologically, topo-II directly transforms a writhe of +1 into a writhe of −1 by converting a right-handed crossing into a left-handed one, whereas topo I can do the same by allowing the DNA to get rid of the extra positive twist (or supercoiling) acquired in the process of changing the sign of the writhe.

We suggest that the topological transformations between local twist and writhe in the chromatin fibre, which we can follow clearly by analysing the reconstitution dynamics *in silico*, are at the basis of the finding that topo-I and topo-II can relax positively supercoiled yeast circular minichromosomes (49) (we note that the authors of (49,50) propose a similar explanation for their results). Also relevant is the recent discovery (54) that the activity of enzymes such as topoisomerase can depend on how the motion of distant DNA regions is constrained—i.e. the rate of topoisomerase action could be reduced due to tethering of the DNA to other proteins. Most importantly, our results provide a strong suggestion that topological considerations are key to the biophysics of chromatin assembly on *linear*, as well as on circular, DNA, at least when the dynamics of local rearrangements and strand collision occur over shorter time scales than those required for the equilibration of the whole chromatin fibre (for a long DNA molecule this will always be the case). Therefore, one may expect that even *in vivo*, when nucleosomes need to be formed, for instance after DNA repair, topological obstacles similar to those leading to the defects analysed in Figures 2 and 3 could arise; indeed, the cell seems to have adopted suitable countermeasures to avoid these (such as the use of chaperones, remodelling factors, etc. (46)).

**Figure 7.**
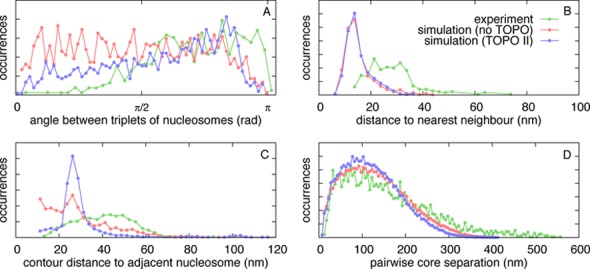
Comparing with experimental measurements extracted from AFM images. Data from (39) is compared with measurements from simulated configurations which have been flattened onto a plane. Simulations were from a 22.1 kbp DNA molecule, using the ΔLk = −1.0 model nucleosomes, for the cases without and with topoisomerase II action, as described in the text. The experiments involved reconstitution of chromatin using DNA molecules with 601 positioning elements separated by linker DNA of length 60 bp (20.4 nm); we compare with the simulations where nucleosome cores were regularly pre-positioned such that the expected linker length is 73 bp (24.8 nm). (A) Distribution of the angle between consecutive triplets of nucleosomes. Experimental data are from Figure 3–13C in (39), which actually show a combination of angle between triplets of nucleosome and the angle between the entry-exit DNA strands where this was clearly visible. (B) Distribution of the straight-line 2D distance between a nucleosome and its nearest neighbour (in 2D, not necessarily one of its neighbours along the fibre). Experimental data are extracted from the AFM image in Figure 3–16A in (39) (reproduced here in Supplementary Figure S4A). (C) Distribution of the contour distance (i.e. along the DNA path) between consecutive nucleosomes along the fibre. Experimental data are from Figure 3–12C in (39); where the DNA is not visible on the AFM image the nucleosome centre–centre distance is used instead. (D) Distribution of the separation of each pair of nucleosomes within a chromatin fibre. Experimental data are extracted from the AFM image in Figure 3–16A in (39) (reproduced here in Supplementary Figure S4A).

The focus of our simulations is on the role of DNA topology and supercoiling in nucleosome formation, and we do not expect them to faithfully reproduce accurately the overall structure of a chromatin fibre, which will depend on DNA sequence and on finer details of the proteins – charge distribution, histone tails etc. – which are not described here. Nevertheless, it is interesting to perform a more quantitative comparison of our final structures with those obtained after *in vitro* reconstitution, and imaged using AFM. Figure 7 shows distributions for several quantities which have been measured from AFM images taken from a recent study (39) where chromatin fibres were reconstituted via salt dialysis using DNA molecules with regularly spaced 601 positioning sequences. Quantities which can be measured are the positions of the nucleosomes (see (39) for details), and where visible, the length of linker DNA between consecutive nucleosomes, and the angle between the entry and exit DNA strands. For comparison the data are shown alongside results taken from simulated configurations flattened onto a 2D plane (as in Figures 2F and 5C); in the experiment the 601 sequences were separated by a regular linker length of 20.4 nm (60 bp) whereas in the simulations the nucleosome cores were pre-positioned with a spacing representing a slightly larger linker length of 24.8 nm (73 bp). Shown in Figure 7A–D, respectively, are (i) the angle between triplets of consecutive nucleosomes; (ii) the distribution of the separation between a nucleosome and its spatial nearest neighbour (not necessarily one of its neighbours along the fibre); (iii) the distribution of DNA contour length between consecutive nucleosomes along the fibres; and finally (iv) the pairwise nucleosome separation distribution.

The AFM data and the simulation show a good match in the angular distribution shown in (i). This level of agreement is encouraging, although it might to some extent be fortuitous, as the experiments measured a combination of angle between nucleosome triplets and entry–exit angles; in our simulations these two quantities have different distributions. The experimentally recorded distributions of nearest neighbour distances (spatial or adjacent along the chain), on the other hand, are quite different from those found in simulations. Interestingly, the mean linker DNA observed in the AFM image is 41.1 nm: considerably larger than the expected 31.4 nm (601 element spacing of 20.4 nm plus 11 nm nucleosome diameter). The most likely reason for this discrepancy and for the broad experimental distributions is that many of the 601 elements did not contain a nucleosome, whereas the simulations shown in Figure 7 started with pre-positioned histones, which favours a more uniform nucleosome coverage within the fibre, and leads to more peaked distributions in Figure 7B and C. It is also known that some nucleosomes may unwrap partially in experiments, either during chromatin reconstitution or due to interaction between the DNA and the positively charged mica surface, see (42) and Supplementary Figure S4E. Another interesting observation is that for both the experimental data and the simulations, the distribution for nearest neighbour separation (Figure 7B) has a peak at a shorter distance than the contour length distribution. This implies that either the DNA is very bent or more likely that the nearest neighbour is not the next nucleosome along the fibre, and reflects the coiled (or zig-zagging) configuration the fibres adopt. Finally, the pairwise nucleosome separation distribution (Figure 7D) shows a good quantitative comparison between experiments and simulations. The fact that the experimental distribution is slightly broader, and the mean slightly larger, may be due to the fact that in simulations we neglect repulsive electrostatic interactions between the DNA beads, which can lead to a straighter linker DNA and hence to a larger internucleosomal distance and overall a more open fibre.

In summary, Figure 7 shows a reasonable agreement between our simulations and AFM images; the main reasons for the discrepancies are that not all nucleosomes form as planned in the experiments. It is notable that the difference between the simulations with and without topo-II is relatively small as regards the distributions presented in Figure 7. Therefore, our results also suggest that when determining the quality of a reconstituted chromatin fibre *in vitro*, digestion experiments such as the one simulated in Figure 6 may be more telling than image analysis.

We close with a comment on the structure found in the *in silico* reconstitution with topo-II (Figure 5). As mentioned above, in our simulations we do not take into account the histone tails, nor do we include a full description of the charges and screening due to salt. Recent simulations of pre-assembled nucleosomes from which the DNA cannot unwrap (15) have shown that at low salt H1 linker histone-free chromatin will adopt an extended beads-on-a-string conformation due to strong electrostatic repulsion between the linker DNA; in physiological salt, much of the repulsion is screened (both by the salt, and the histone H3 tails), and balanced out by a histone tail mediated attraction between the nucleosomes. This results in a more zig-zagging conformation where the nucleosomes most closely interact with their next-to-nearest neighbour nucleosome along the fibre (15). Interestingly our fibres also exhibit these features (Figure 5A and C).

[We note here that the zig-zag appearance of a fibre can be quantified by looking at which nucleosomes are closest together in space, i.e., is it usually the next nucleosome along the fibre, or, for example, the next but one? For fibres assembled without the action of topological enzymes, nearest neighbours are most often consecutive nucleosomes. In the high density case, adding topo-II yields fibres where nearest neighbours are often the next-but-one consecutive nucleosomes along the fibre – it resembles a zig-zag. The effect is strengthened when configurations are flattened onto a 2D plane, but weakened in the case of a lower nucleosome density (longer linker length).]

## CONCLUSION

In conclusion, we have presented *in silico* chromatin reconstitution experiments in which we have attempted, for the first time, to self-assemble a chromatin fibre from a solution containing naked DNA and histone proteins. Besides reproducing the formation of aggregates in synthetic chromatin made up by DNA and spherical (uniformly charged) nanoparticles (Figure 1), our simulations show that when modelling the assembly of a chromatin fibre from histone octamers and DNA, several defects arise, often associated with histone clustering (Figures 2 and 3).

The defect found most commonly *in silico* is where the DNA only partially wraps around the octamer, and further wrapping is inhibited by a local entanglement which cannot be resolved without strand crossing (Figure 3A and B). Remarkably, allowing for topo-II-like strand crossing leads to much more ordered structures in our simulations, resembling a textbook 10-nm chromatin fibre (Figures 4 and 5). A similar increase in the quality of the reconstituted fibre can be obtained by using enzymes which allow twist relaxation (e.g. by nicking the DNA), such as topo-I. A valuable message which can be taken from this work is that topological defects may often be present in chromatin fibres reconstituted *in vitro*, and that these might not be evident from an analysis of AFM images (Figure 7), whereas a nuclease digestion experiment, such as in (49), might be more telling.

In the future our *in silico* approach to chromatin self-assembly could be combined with models which have been used to study higher order chromatin structures (7–14), i.e. combining a core particle from which the DNA can unwrap, with finer details such as sequence heterogeneity (21), or the charged histone tails which can lead to bridging-mediated interactions between nucleosomes (55–57), and compaction into higher order structures by histone protein H1 (1,2). It may then be possible to model, for example, some aspects of chromatin reconstitution following DNA repair, or the structure of a eukaryotic gene undergoing transcription, where a region of positive supercoiling, where topo-II is enriched, is generated ahead of the polymerase (58,59).

## SUPPLEMENTARY DATA

Supplementary Data are available at NAR Online.

SUPPLEMENTARY DATA
