# Supplementary material for: Topological constraints strongly affect chromatin reconstitution in silico
Source: Nucleic Acids Res. 2014 Nov 28;43(1):63–73. doi: 10.1093/nar/gku1085 (PMC4288149; doi:10.1093/nar/gku1085)
Supplement: SUPPLEMENTARY DATA [file supp_43_1_63__index.html]

Topological constraints strongly affect chromatin reconstitution in silico — SUPPLEMENTARY DATA 

# Topological constraints strongly affect chromatin reconstitution *in silico*

## SUPPLEMENTARY DATA

**Files in this Data Supplement:**

- SUPPLEMENTARY DATA
- SUPPLEMENTARY DATA
- SUPPLEMENTARY DATA
- SUPPLEMENTARY DATA
- SUPPLEMENTARY DATA
