# Supplementary material for: Topological constraints strongly affect chromatin reconstitution in silico
Source: Nucleic Acids Res. 2014 Nov 28;43(1):63–73. doi: 10.1093/nar/gku1085 (PMC4288149; doi:10.1093/nar/gku1085)
Supplement: SUPPLEMENTARY DATA [file supp_gku1085_nar-02096-f-2014-File004.pdf]

Topological constraints strongly affect chromatin reconstitution *in silico*

Supplementary Data

C. A. Brackley,[1] J. Allan,[2] D. Keszenman-Pereyra,[3] and D. Marenduzzo[1]

[1] *SUPA, School of Physics & Astronomy, University of Edinburgh, Edinburgh, EH9 3FD, UK*
[2] *Institute of Cell Biology, University of Edinburgh, Edinburgh, EH9 3BF, UK*
[3] *Edinburgh Genomics, Ashworth Laboratories, University of Edinburgh, Edinburgh, EH9 3FL, UK*

I. **Supplementary Methods.**
II. **Reconstitution via salt dialysis: in vitro and in silico.**
III. **Initial conditions**
IV. **Simulated surface deposition**
V. **Classifying nucleosome wrapping**
VI. **Modelling the action of topological enzymes**
VII. **Simulated Nuclease Digestion**
VIII. **Supplementary Figures**
IX. **Supplementary Table**
X. **Supplementary Movie Captions**
XI. **References**

I. SUPPLEMENTARY METHODS

To perform the molecular dynamics (MD) simulations we use standard techniques via the LAMMPS (Large-scale Atomic/Molecular Massively Parallel Simulator) software package (1). We coarse grain the system by treating the DNA as a bead-and-spring polymer, where each bead is a rigidly connected group of spheres. Proteins (nanoparticles or nucleosome cores) are also modelled as spheres, or rigidly connected groups of spheres. We refer to each nanoparticle, nucleosome core, or DNA bead as a "rigid body", and to each constituent sphere as an "atom". LAMMPS was run in Brownian dynamics (BD) mode, where a molecular dynamics algorithm is used with a stochastic thermostat, which models the thermal fluctuations and viscosity of an implicit solvent. For computational efficiency hydrodynamic interactions are neglected (see also the discussion in the section on the mapping to physical units). Using this coarse grained approach — where molecules are represented by only a small number of component spheres — allows our simulations to cover much larger length and time scales than studies which include greater (e.g. atomistic) detail (2–5).

A. Brownian dynamics simulations

Translational motion of the ith rigid body evolves according to the Langevin equation

$$m_i \frac{d^2\mathbf{r}_i}{dt^2} = \mathbf{F}_i - \gamma_i \frac{d\mathbf{r}_i}{dt} + \sqrt{2k_BT\gamma_i}\boldsymbol{\eta}_i(t), \tag{S1}$$

where $\mathbf{r}_i$ is the position of the centre of mass of the body which has total mass m_i; $\mathbf{F}_i$ is the net force on the body, γ_i is the friction due to the solvent, and $\boldsymbol{\eta}(t)$ is a vector representing random uncorrelated noise, such that $\langle\eta_\alpha(t)\rangle = 0$, $\langle\eta_\alpha(t)\eta_\beta(t')\rangle = \delta_{\alpha\beta}\delta(t-t')$. The kth constituent atom of body i is at position $\mathbf{r}_{ik}$, and the net force on each body is the sum of forces on each of these atoms, i.e. $\mathbf{F}_i = -\sum_k \nabla_{ik} U$, where $\nabla_{ik} \equiv \partial/\partial\mathbf{r}_{ik}$. U is the potential due to interactions between all atoms; there is no interaction between atoms within the same rigid body. Rotation is described by a similar Langevin equation, but with a rotational friction γ_R, and the force term

replaced by a torque about the centre of mass the body $T_i = \sum_k (\mathbf{r}_i - \mathbf{r}_{ik}) \times (-\nabla_{ik} U)$.

Below we describe the interaction potentials used in the DNA model and the various protein models. We use phenomenological potentials (e.g. truncated Lennard-Jones) since these offer a significant improvement in computational efficiency over more realistic potentials such as Debye-Hückel. This choice means that we do not consider any additional electrostatic repulsion between atoms, but since at physiological salt concentrations the Debye screening length is of order 1 nm (and is shorter for higher salt concentrations such as the conditions during reconstitution experiments), we do not expect any significant effect on our results.

B. The DNA model

Each bead in the polymer represents $\sigma = 2.5$ nm of DNA (~ 7.4 bp), and consists of a core sphere (diameter σ) and a "patch" (diameter $0.2\ \sigma$) which sits with its centre a distance $0.4\ \sigma$ from the core sphere centre, such that it does not protrude from the core surface; the patch allows us to account for the torsional rigidity of the polymer. A schematic representation is given in Supplementary Fig. S1. This description is appropriate for twistable open polymers; it would need to be refined to describe e.g. a supercoiled DNA loop.

The core sphere of the ith bead in a chain is connected to that of the $i+1$th with a finitely extensible non-linear elastic (FENE) spring given by the potential

$$U_{\mathrm{FENE}}(r_{i,i+1}) = U_{\mathrm{WCA}}(r_{i,i+1}) - \frac{K_{\mathrm{FENE}} R_0^2}{2} \log\left[1 - \left(\frac{r_{i,i+1}}{R_0}\right)^2\right], \tag{S2}$$

where $r_{i,i+1} = |\mathbf{r}_i - \mathbf{r}_{i+1}|$ is the separation of cores of the i and $i+1$th beads. Here the first term represents a hard steric interaction which prevents adjacent beads from overlapping (the Weeks-Chandler-Andersen potential – see Eq. (S3) below), and the second gives the maximum extension of the bond, R_0. Throughout we use $R_0 = 1.6\ \sigma$, and set the bond energy $K_{\mathrm{FENE}} = 30\ k_BT$.

Steric interactions between non-adjacent DNA beads are also given by the Weeks-Chandler-Andersen potential

$$U_{\mathrm{WCA}}(r_{ij}) = 4k_BT\left[\left(\frac{d_{ij}}{r_{ij}}\right)^{12} - \left(\frac{d_{ij}}{r_{ij}}\right)^{6}\right] + k_BT \tag{S3}$$

for $r_{ij} < 2^{1/6} d_{ij}$, and $U_{\mathrm{WCA}}(r_{ij}) = 0$ otherwise. Here d_{ij} is the mean of the diameters of the two atoms, i.e. for two DNA beads $d_{ij} = \sigma$.

The bending rigidity of the polymer is introduced via a Kratky-Porod potential for every three adjacent DNA beads

$$U_{\mathrm{BEND}}(\theta_B) = K_{\mathrm{BEND}}\left[1 - \cos(\theta_B)\right], \tag{S4}$$

where θ_B is the angle between the three beads as shown in Supplementary Fig. S1A, and K_{BEND} is the bending energy. The persistence length in units of σ is given by $l_p = K_{\mathrm{BEND}}/k_BT$; the persistence length for naked DNA is well characterised, and we use $l_p = 20\ \sigma = 50$ nm.

Finally, the torsional rigidity of the polymer is provided by a second angle potential and a dihedral potential. The angle potential is given by

$$U_{\mathrm{TORS}}(\theta_T) = 100 k_BT\left(\theta_T - \frac{\pi}{2}\right)^2, \tag{S5}$$

where θ_T is the angle between the ith bead patch, the ith bead core, and the $i+1$th bead core, as shown in Supplementary Fig. S1B; this merely acts to keep the patch such that the line connecting a core to a patch points perpendicular to the axis of the polymer. The dihedral potential is given by

$$U_{\mathrm{DIH}}(\phi) = K_{\mathrm{DIH}}\left[1 + \cos(\phi + \pi)\right], \tag{S6}$$

where ϕ is the angle between the plane defined by the centres of the ith bead patch, ith bead core, and $i+1$th core, and the line connecting the $i+1$th core and patch (Supplementary Fig. S1C). This potential constrains rotation of DNA beads about the axis of the polymer. The torsional rigidity of DNA is not precisely known, but a consensus value is 3×10^{-19} erg cm (6–8); in our simulation units this corresponds to $K_{\mathrm{DIH}} = 29.7\ k_BT$.

C. Spherical nucleosome cores

In Fig. 1 in the main paper we present a simulation of DNA interacting with spherical nucleosome cores, similar to experiments using gold plated nanoparticles (9, 10). We model these nanoparticles as single spheres (11–13) of diameter $2.7\ \sigma$ (~ 6.75 nm), which interact with each other via the Weeks-Chandler-Andersen potential given in Eq. (S3) — i.e. there is only a steric repulsion. They interact with the DNA beads (with the core of the bead - blue in figures) via a truncated shifted Lennard-Jones potential

$$U_{\mathrm{LJ\ cut}}(r_{ij}) = \begin{cases} U_{\mathrm{LJ}}(r_{ij}) - U_{\mathrm{LJ}}(r_{\mathrm{cut}}) & r_{ij} < r_{\mathrm{cut}}, \\ 0 & \text{otherwise}, \end{cases} \tag{S7}$$

with

$$U_{\mathrm{LJ}}(r) = 4\epsilon'\left[\left(\frac{d_{ij}}{r}\right)^{12} - \left(\frac{d_{ij}}{r}\right)^{6}\right],$$

where r_{cut} is a cut off distance, and r_{ij} and d_{ij} are the separation and mean diameter of the two atoms respectively. This leads to an attraction between the spheres and the DNA cores if their centres are within a distance r_{cut}. Here ϵ' is an energy scale, but due to the second term in Eq. (S7) this is not the same as the minimum of the potential, which for clarity we denote ϵ (and it is this which we refer to as the interaction energy). For the spherical nanoparticles we use $r_{\mathrm{cut}} = 2.2\ \sigma$. In Fig. 1 the interaction energy is $\epsilon = 4.3\ k_BT$, but similar results are obtained for different values (in Supplementary Fig. S2A, $\epsilon = 12\ k_BT$).

D. Nucleosome cores with helical binding path

The nucleosome core model used for the majority of the simulations in this work consists of two central spheres of diameter $2.7\ \sigma$, and 20 smaller spheres of diameter $0.2\ \sigma$, which represent the strong DNA binding sites corresponding to positively charged patches on the histone octamer surface. These are arranged in a helix making 1.7 turns about the central spheres — see Fig. 2B and C.

The large central spheres interact with DNA atoms only sterically, using the WCA potential given in Eq. (S3). The small spheres also interact with the core of the DNA beads via this potential, but interact with the DNA patches via the shifted LJ potential given in Eq. (S7) with $r_{\rm cut} = 0.6\ \sigma$. This means that there is an attractive region extending $0.5\ \sigma$ from the side of each DNA bead. There are only steric interactions between different nucleosome cores. This model has some similarities to recent work on force induced nucleosome unravelling (14).

In our model the nucleosome cores interact only with the patch on each DNA bead; in reality the strongest interactions between the histone proteins and the DNA are where arginine chains from the histone proteins sit in the minor grove of the DNA where it faces the octamer (15); the strong binding region on the DNA might therefore be a continuous helical path along the length of the molecule. The coarse graining in the model necessitates that the nucleosome cores only interact with discrete patches on the DNA beads. This means: (i) that in our model the probability of the nucleosome cores binding to DNA segments far away along the contour from the initial position of the core might be reduced compared to reality, (ii) that our DNA will only wrap with the side with the binding patches facing the core, and (iii) that it may be more difficult than in reality for the DNA to wind around the core with any additional twist (this could potentially hinder the nucleosome diffusion mechanism proposed in (16, 17)). We do not however expect these shortcomings to qualitatively affect our reconstitution results; furthermore discrete binding patches could be thought to represent sequence motifs which have a high affinity for histone binding (e.g. since the minor groove is more likely to face the octamer at AT rich segments, there is a preference for wrapping with a particular side facing the octamer (15, 18)).

Note that in a real nucleosome, the minor groove of the 49.6 nm of DNA which wraps around the histone octamer is facing the proteins at 14 locations — i.e. there are 14 binding sites (15). Since we are using a coarse grained DNA bead of 2.5 nm, then 20 of these are required to wrap the core, so our nucleosomes have 20 binding patches.

The interaction energy between the DNA and the nucleosome cores is set to $\epsilon = 12\ k_BT$ per binding site, meaning that the binding energy for a correctly wrapped nucleosome is 240 k_BT. This is offset by the $\sim 60\ k_BT$ cost of bending 1.7 turns of DNA around a diameter of 10 nm, and leads to a net bound state energy of 180 k_BT. This choice was made because it is the smallest binding energy for which a correctly wrapped nucleosome is stable on simulation time scales; it is close to, albeit somewhat larger than, the value of around $85\ k_BT$ which is indicated by competitive protein binding experiments (17, 19). Such a strong binding energy means that if a core is pre-positioned such that one of its binding sites is bound to a DNA bead, then any movement of the core along the DNA before wrapping is very slow. In a salt dialysis experiment the H3-H4 tetrameters will initially have a very weak interaction with the DNA — increasing as the salt concentration reduces; it is likely that it is during this early phase of the reconstitution that any sequence derived positioning takes place. Our simulations therefore capture the dynamics of a later phase of the dialysis experiment, where the tetramers are already positioned.

E. Nucleosomes Store DNA Twist

As detailed in the main text, it is known from the x-ray crystal structure of the nucleosome, that the DNA is twisted as it wraps around the histone octamer. We can easily incorporate this into the model by introducing an angle between the binding patches on adjacent DNA beads. When the DNA wraps around the core, it must then twist in order for the DNA binding patches to line up with those on the core. This is achieved my a slight modification to Eq. (S6), as follows

$$U_{\rm DIH}(\phi) = K_{\rm DIH}\left[1 + \cos(\phi_0 + \phi + \pi)\right].$$

Our nucleosomes give rise to -1.75 units of writhe; to give an overall linking number change of only ΔLk=-1 therefore requires $+0.75$ units of twist to be stored in the 20 beads which bind to the core. A value $\phi_0 = 14.2^\circ$ in the above potential will achieve this. This force field is used in Figs. 5-7. and Supplementary Figs. S6-S9.

F. Simulation units and mapping to physical units

We use energy units of k_BT, length units of $\sigma = 2.5$ nm, and set the simulation mass units by choosing $m = 1$ for each DNA bead (core plus patch). A DNA segment of length 2.5 nm has a mass of $\sim 8 \times 10^{-24}$ kg, whereas a histone octamer has mass $\sim 1.67 \times 10^{-22}$ kg, approximately 20 times larger, so we choose a mass of $20\ m$ for the model nucleosome cores (total mass of all constituent atoms).

The definition of the simulation time unit $\tau = \sigma/\sqrt{mk_BT}$ follows naturally from the above, but we map it to physical time units by considering the Brownian time for each body, defined as the time it takes to diffuse across its own diameter d, i.e. $\tau_B = d^2/D$. An exact mapping of simulation units to physical ones is not possible, since for a 8×10^{-24} kg, 2.5 nm sphere in a fluid of 1 cP viscosity the inertial time (the time it takes to lose velocity information, defined as $\tau_{\rm in} = m/\gamma$) is $\tau_{\rm in} \approx 0.0016\tau_B$; this separation of time scales would require infeasibly long simulation times, so instead we use $\tau_{\rm in} = 0.25\tau_B$. This means the bodies in our simulation have more inertia than in reality, but we are still in the over-damped regime where $\tau_{\rm in} < \tau_B$.

The diffusion constant for each body is set via the friction γ through the Einstein relation $D = k_BT/\gamma$. We use

$\gamma = 2\ m/\tau$ for the DNA beads of diameter $d = 1\ \sigma$. To map to physical time scales we consider the Brownian time for each body, defined as the time it takes to diffuse across its own diameter d, i.e. $\tau_B = d^2/D$, and the viscosity ν of the implicit fluid as given by the Stokes relation $\gamma = 3\pi\nu d$ for a sphere of diameter d. This leads to a simulation viscosity $\nu = 2/3\pi$ and DNA bead Brownian time $\tau_B = 2\ \tau$ (simulation units). A sphere of diameter 2.5 nm in an aqueous fluid (viscosity 1 cP) has $\tau_B \approx 35.6$ ns, meaning one simulation time unit is equivalent to 17.8 ns. For nucleosome cores of diameter $d = 2.7\ \sigma$ we need to choose $\gamma = 5.4\ m/\tau$ to give the same viscosity. For rotation, we make the approximation that all bodies rotate like spheres, and use the Stokes relation for rotational friction $\gamma_R = \pi\nu d^3$.

While our Brownian dynamics scheme correctly incorporates translational and rotational drag of single spheres and rigid bodies, as previously mentioned it does not include hydrodynamic interactions between beads, mediated by the underlying solvent. This can quantitatively affect the time-scale for polymer equilibration and torsional relaxation, although the basic fact that the former is longer for a sufficiently long polymer will remain true.

G. Simulation details

In each simulation we have $L = 3010$ or $L = 6020$ DNA beads (corresponding to 22 kbp or 44 kbp respectively) and 100 nucleosome cores, and for each set of results we ran 10 separate simulations in parallel using different seeds for the LAMMPS random number generator, and different initial configurations (see section III below for further details). Each set of simulations was run on 10 cores using AMD Opteron^{TM} 6168, 1900 MHz Processors. For the $L = 3010$ sets of simulations equilibration runs of 10^8 time steps (1 time step=10^{-3} time units) took approximately 17 hours (170 total CPU hours) with productions runs of 1.6×10^7 time steps taking approximately 27 hours (270 total CPU hours). For the $L = 6020$ sets of simulations productions runs of 1.6×10^7 time steps took approximately 54 hours (540 total CPU hours).

II. RECONSTITUTION VIA SALT DIALYSIS: IN VITRO AND IN SILICO.

The most common method of *in vitro* chromatin reconstitution is salt dialysis (12, 13) where purified histone proteins and DNA are mixed in a buffer with a very high salt concentration (typically 2 M NaCl; for full details and a review see e.g. (20)). The salt is then slowly dialysed out of the system over a period of 30 to 36 hours, until physiological concentrations are reached. A naive mixing of histones and DNA at physiological salt (or a rapid dilution from high to low salt) results in large protein-DNA aggregates which drop out of solution; histone octamers are not stable in the absence of wrapping DNA at physiological salt.

At 2 M NaCl the histone octamer is a stable oligomeric complex. If the salinity is reduced to around 1 M NaCl the octamers become unstable, and break into tetramers consisting of two of each of the H3 and H4 histones, and two dimers containing one each of the H2A and H2B histones. The usefulness of the dilution assay comes form the fact that the tetramers start to interact with the DNA at a higher salt concentration than the dimers, so that the nucleosome forms in two stages. First the tetramers bind to the DNA, and a single turn is wrapped (sequence specificity in binding is likely to be most important during this stage); then, as the salt concentration is decreased further, dimers start to interact with the tetramer-DNA complexes. Complete assembly is achieved by 0.6 M NaCl, and the assembled fibres remain stable if transferred to a lower (physiological) concentration salt buffer.

In our simulations we mimic the dilution assay by switching on interactions between the nucleosome cores and the DNA in two stages. (A slow continuous change of the interactions over the time scales of the *in vitro* salt dilution would lead to infeasibly long simulations.) For the first 0.1 ms of each simulation only the first turns' worth of binding sites on the nucleosome cores are able to interact with the DNA — i.e. during this phase of the simulation the model cores represent a 2H3-2H4 tetramer. These binding site are coloured yellow in figures. In the second half of the simulation the full 1.7 turns of binding sites are switched on — the dynamics of this second stage represent two H2a-H2b dimers joining the complex and wrapping the remaining DNA. The second set of binding sites are coloured red in figures.

III. INITIAL CONDITIONS

For the simulations of the nucleosome cores with helical binding path we consider two different initial conditions: cores pre-positioned at regular intervals along the contour of the DNA (as in Figs. 2 and 4), and cores positioned at random locations along the DNA (in Supplementary Fig. S6). The former case might correspond to using a DNA molecule with a sequence which contains strong nucleosome positioning elements at regular intervals (for example as is used in Refs. (21),(22) and (23) to produce nucleosomal arrays), whereas the latter could result form a sequence with weak positioning.

In both cases we start with a DNA molecule which has an equilibrium coil-like configuration obtained in the absence of nucleosome cores. This is achieved by starting with a random walk configuration and initially using "soft" harmonic potentials instead of the LJ and FENE interaction described above; this generates self-avoiding configurations while avoiding numerical issues. The equilibration is then run for at least 8×10^4 time units (1.5 ms). Nucleosome cores are then positioned at the regular or randomly selected locations along the DNA molecule, such that the central binding site in the helix is in contact with a DNA bead patch. For the random positions, DNA beads are chosen at random, but are rejected if they fall within 10 beads of another nucleosome position, as this would be disallowed by steric interactions.

For the spherical nanoparticle simulation shown in Fig. 1 a similar equilibrium DNA configuration is obtained, and

the spheres are distributed randomly through the system. For Supplementary Fig. S2B the spheres are pre-positioned randomly along the DNA molecule in the same way as above.

IV. SIMULATED SURFACE DEPOSITION

In order to better compare our simulation with images from atomic force microscopy or electron microscope imaging we have simulated the deposition of our three dimensional self-assembled fibres onto a two dimensional surface. This was done by subjecting DNA beads to a "wall" potential

$$U(z)_{\rm wall} = K_{\rm wall} z^3, \qquad \text{(S8)}$$

where z is the distance of the DNA bead from the wall and we set the energy $K_{\rm wall} = 100\ k_BT$. Configurations were taken from the end of the ~ 0.3 ms runs, and two walls aligned in the x-y plane were introduced such that they just enclosed the assembled fibre. The separation of the two walls was then reduced over a further simulation run of 0.14 ms until they were 10 nm apart. This allowed time for the fibre to rearrange as it was flattened onto the plane. Figures 2F and 4C show self-assembled fibres which have been treated in this way.

V. CLASSIFYING NUCLEOSOME WRAPPING

Supplementary Table S1 shows the proportion of nucleosome cores which are found to be correctly wrapped, partially wrapped or forming loops etc. for the various different simulations. Correctly wrapped nucleosomes are easily identified by checking that each of the 20 binding sites on the model nucleosome core is in contact with the binding site on a DNA bead, and that these DNA beads are consecutive along the polymer (we define "in contact" as the centres of the patches having a separation less than 1.25 nm). We also classify nucleosome cores as correctly wrapped if only the last binding site at either end of the helical path of binding sites remains unbound to a DNA bead; this corresponds to a nucleosome in which up to 8 bp of DNA has become unravelled at either end — such breathing of nucleosomes has been observed via FRET studies (24, 25).

Nucleosome cores in which only some of the binding sites are bound to DNA (and those which are, are bound to consecutive DNA beads) are classified as partially wrapped (Figs. 3A and B). As detailed in the main paper this often results when there are topological barriers to complete wrapping. In theory such barriers can always resolve themselves given enough time (however, according to the Rouse theory of polymer dynamics (26) the time needed will scale with the square of the length of the DNA molecule); in practice, unprotected nucleosome binding sites can interact with other DNA segments, forming other metastable structures. We classify any nucleosome which is bound to DNA beads which are not consecutive along the polymer as "forming DNA loops" (Figs. 3C and 3D). Such loops may be small bubbles, or large scale loops where the nucleosome core joins DNA beads which are distant along the DNA contour. Due to the high binding affinity, when a partially wrapped nucleosome becomes a loop forming nucleosome, this is typically irreversible on simulation time-scales.

DNA can also wrap around the cores in a right-handed fashion, but in this configuration fewer bonds are made than when the DNA follows the left-handed helical binding path. In our simulations these can also often occur due to topological barriers to left-handed wrapping; the wrapped DNA forms an effective barrier to further interaction with other DNA segments. The reversomes which have been observed both *in vitro* and *in vivo* (27) differ from the right-handed wrappers in our simulations in that they involve an alternative structure of histone octamer; nevertheless one can hypothesise that the formation of this less stable octamer could be driven by topological constraints on the DNA.

Finally in several simulations we find nucleosome cores which have escaped from the DNA. This occurs when a nucleosome escapes from the DNA at the start of a simulation before any wrapping takes place, and can be as a result of initial configurations where there is only a weak contact between the nucleosome and the DNA.

VI. MODELLING THE ACTION OF TOPOLOGICAL ENZYMES

As detailed in the main paper, many of the defects seen in our simulated chromatin fibres arise due to topological constraints on the DNA. In the cell there are a number of specialized enzymes which can relax these topological constrains; one of these, topoisomerase II, acts by using ATP to generate a double strand break in a DNA molecule, allowing the broken strand to pass around another, before rejoining the broken ends (28).

In the simulations we mimic the action of topo-II by replacing the LJ interaction between non-adjacent DNA beads with a soft potential

$$U_{\rm soft}(r) = \begin{cases} K_{\rm soft}\left[1+\cos\left(\frac{\pi r}{r_{\rm cut}}\right)\right] & r < r_{\rm cut}, \\ 0 & \text{otherwise}, \end{cases} \qquad \text{(S9)}$$

where r is the separation of the two beads, and we use a cut off $r_{\rm cut} = 2^{1/6}\sigma$. This means that the DNA is still preferentially self-avoiding, but there is a reduced energy barrier to strand passing of magnitude $K_{\rm soft}$. Supplementary Fig. S5A shows how the simulation progresses when the topoisomerase II action is introduced for a duration of 0.1 ms at the start of the second phase of the simulation (where all DNA-nucleosome core interactions are switched on), for various values of $K_{\rm soft}$. By looking at the mean amount of DNA wrapped around each nucleosome core at the end of the simulation, one can see that there is a transition where for $K_{\rm soft} \lesssim 3$ the self-assembly is significantly improved (i.e. the energy barrier is sufficiently low that the forces induced as the DNA wraps up on the cores gives rise to strand passing); in Fig. 4 Supplementary Fig. S8 and Supplementary Table S1 a value $K_{\rm soft} = 2\ k_BT$ is used.

Topological constraints can also be removed by relaxing the torsional rigidity of the DNA (i.e. by reducing the value of $K_{\mathrm{DIH}} \rightarrow 0$ to allow rotation of the beads about the polymer axis). This is equivalent to introducing nicks into the DNA molecule, and is related to the action of type I topoisomerase. Supplementary Fig. S5B shows how the simulation progresses when K_{DIH} is reduced to various values for a duration of 0.1 ms at the start of the second phase of the simulation (where all DNA-nucleosome core interactions are switched on). Supplementary Fig. S8 and Supplementary Table S1 include results from simulations where K_{DIH} is temporarily reduced to 4 k_BT.

VII. SIMULATED NUCLEASE DIGESTION

A common experimental probe of chromatin fibres is nuclease digestion. The fibre is treated with an enzyme such as micrococcal nuclease, which preferentially digests the DNA in the linker regions between nucleosomes. The lengths of the resulting fragments are resolved by gel electrophoresis; a typical gel shows bands (reflecting the periodicity of the nucleosome positioning) from fragments which contained different numbers of nucleosomes. By treating with the enzyme for an extended time an extreme digestion can be performed; here all DNA which is not wrapped in nucleosomes is digested, and the resulting gel shows a strong band only at ~ 147 bp.

To simulate the nuclease digestion (29) on our *in silico* chromatin fibres we first extract from the simulations a list of all DNA beads which are bound to a nucleosome core (this means that there is an implicit approximation that no further relaxation and rearrangement of the DNA and nucleosomes can occur during the digestion). From the remaining DNA we randomly pick beads to be digested, such that on average the DNA is cut every 294 bp (this corresponds to a probability of 0.025 that any given bead is digested). Any fragments which do not contain DNA beads which are bound to nucleosome cores are removed (*in vitro* these fragments would likely be further digested by the nuclease). A histogram of the lengths of the remaining fragments (Supplementary Fig. S9) is used to construct the gel pattern (Fig. 5). Each set of results consists of 10 simulated fibres (each with 100 nucleosome cores), and for each of these we repeat our random cutting algorithm 100 times (i.e. the *in silico* digestion is performed on 1000 simulated fibres). Supplementary Figs. S9B and D show histograms for the regularly spaced high and low nucleosomal density fibres respectively. For the high nucleosomal density (shorter linker length between nucleosomes) case, distinct peaks are seen for the case with topoisomerase; without topoisomerase DNA looping etc. smears out the clear ladder. For the low density case, the linker length is long enough that the distribution of lengths for fragments containing one nucleosome overlaps with that of fragments containing two nucleosomes, etc., so individual peaks cannot be seen. For the case of random spacing (random linker lengths) no peaks can be seen (data not shown); although one would expect that for a large number of fibres with random nucleosome spacing, end effects would give rise to a narrowed distribution of linker lengths (see for example Ref. (29)), this is not seen for only 10 different simulated fibres.

In supplementary Figs. S9A and C we also show the case of a digestion experiment take to the extreme – where all DNA beads which are not bound to the nucleosome cores are removed. The resulting histograms show a strong peak at 147 bp corresponding to correctly wrapped nucleosomes; for the simulations without topoisomerase, peaks are also seen for smaller fragments, which correspond to partially wrapped nucleosomes etc. These results might not be easily observed in reality, as during the coarse of the digestion experiment, partially wrapped nucleosome defects might become resolved as the digested ends of the DNA are presumably free to rotate.

VIII. SUPPLEMENTARY FIGURES

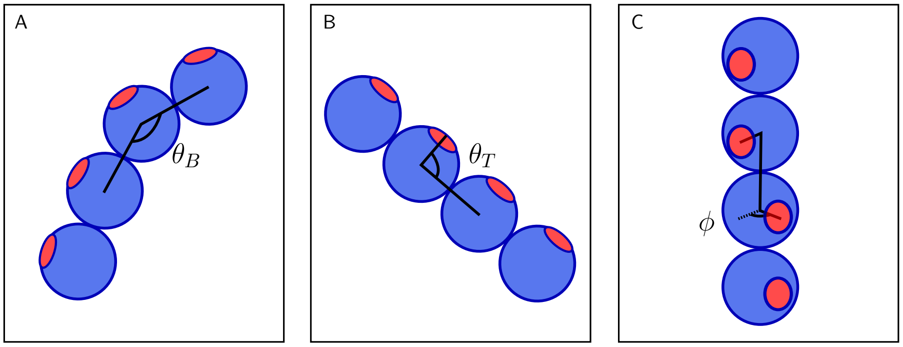

Supplementary Figure S1: DNA modelled as a bead-and-spring polymer with torsional rigidity Schematic diagrams of DNA beads and the angles used in the force field (A) Diagram showing the angle involved in the bending potential. (B) Diagram highlighting the θ_T angle ensuring that the patches remain on the "side" of the DNA chain (see main text). (C) Diagram showing the dihedral angle used to model the torsional rigidity of DNA. The contributions to the potential corresponding to (A-C) are given in Eqs. (S4-S6).

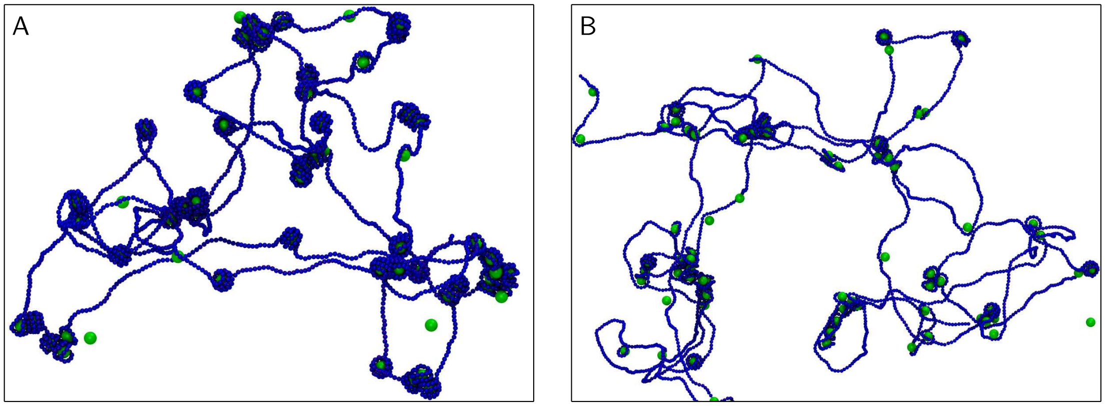

Supplementary Figure S2: Clusters of spheres form under many different conditions. When spheres are used as a model for a histone octamer, the resulting structure contains clusters of spheres. This holds both for different DNA-sphere interaction energies (panel A is from a simulation with interaction energy $\epsilon = 12\ k_BT$, compared to $\epsilon = 4.3\ k_BT$ in Fig. 1), and for initial conditions where the spheres are pre-positioned at regular positions along the DNA (panel B).

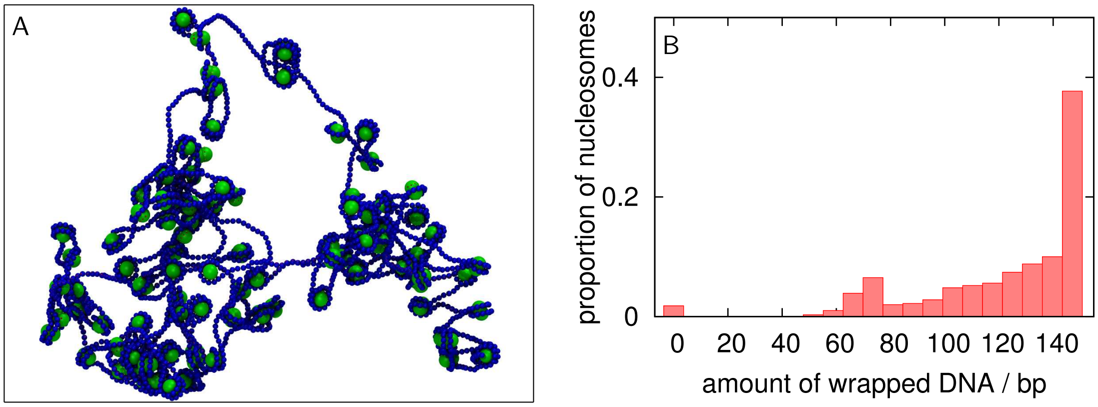

Supplementary Figure S3: Single step reconstitution. A single step reconstitution (where all binding sites on the nucleosome cores interact with the DNA for the entire duration of the simulation) performs less well than the two step process (as used for Figs. 2-4; this mimics the salt dilution). Panel A shows a typical configuration after 0.3 ms, with B showing a histogram of the amount of DNA wrapping each of 1000 nucleosomes (100 cores in each of 10 different simulations). Initial conditions were to pre-position the cores at regular intervals along an equilibrated DNA.

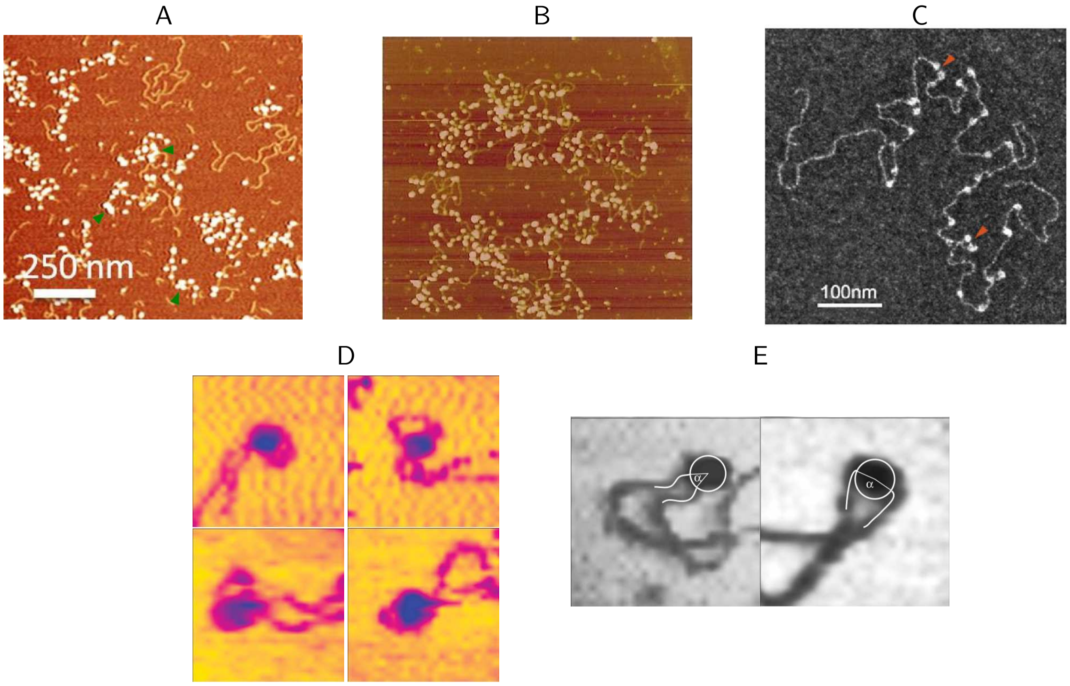

Supplementary Figure S4: Microscopy images of reconstituted nucleosomes showing defects with similar morphology to those identified in simulations (Fig. 3). (A) Atomic microscope image of nucleosomes reconstituted via salt dialysis onto an array of 601 positioning sequence. *Reproduced with permission from Fig. 3-16A in* (30). Arrows highlight clusters of nucleosomes which could represent defects such as those in Fig. 3D and F. This figure shows a zoomed region of the imaged used to generate the data in Figs. 6B and D. (B) Similar image to that shown in Fig 2D, but a greater number of nucleosomes are bound to the DNA and fewer clusters are observed. Nucleosomes have been reconstituted onto a long (106 kbp) circular, supercoiled DNA molecule. *Reproduced with permission from Fig. 4 in* (31). (C) Electron microscope image of nucleosomes reconstituted via salt dialysis onto a shorter (9 kbp) DNA molecule (see Ref. (32) for details); arrows show clusters of close packed nucleosomes which may be as a result of defects such as those in Fig. 3D and F. *Reprinted from Fig. 1A in Mol. Cell, Vol 27, A Bancaud et al., Nucleosome Chiral Transition under Positive Torsional Stress in Single Chromatin Fibers, Pages 135–147, Copyright (2007), with permission from Cell Press* (33). (D) SFM images of nucleosomes reconstituted via salt dialysis onto negatively supercoiled pUC18 DNA (absorbed onto mica from 100 mM NaCl - see Ref. (34) for details); these nucleosomes formed at superhelical loop crossings, and may represent intra-nucleosomal loops. This is where four DNA strands emerge from the nucleosome, and is similar to the defect shown in Fig. 3C. *Reprinted from Fig. 4 in J. Mol. Biol., Vol 345, M Bussiek et al., DNA-loop Formation on Nucleosomes Shown by in situ Scanning Force Microscopy of Supercoiled DNA, Pages 695–706, Copyright (2005), with permission from Elsevier* (34). (E) SFM images of nucleosomes with the linker opening angle indicated. This gives a measure of the conformation of the nucleosome, and could represent different amounts (or handedness) of wrapping. This may be the result of defects such as those in Fig. 3A and B. *Reprinted from Fig. 6 in J. Mol. Biol., Vol 345, M Bussiek et al., DNA-loop Formation on Nucleosomes Shown by in situ Scanning Force Microscopy of Supercoiled DNA, Pages 695–706, Copyright (2005), with permission from Elsevier* (34). With the exception of (C), each of these images shows a possible defect formed on a topologically constrained DNA molecule (i.e. a loop). One could also think of the *in silico* DNA molecules as begin effectively topologically constrained: whilst the ends can move freely, not enough time has passed during the simulations for large scale topological rearrangement to occur.

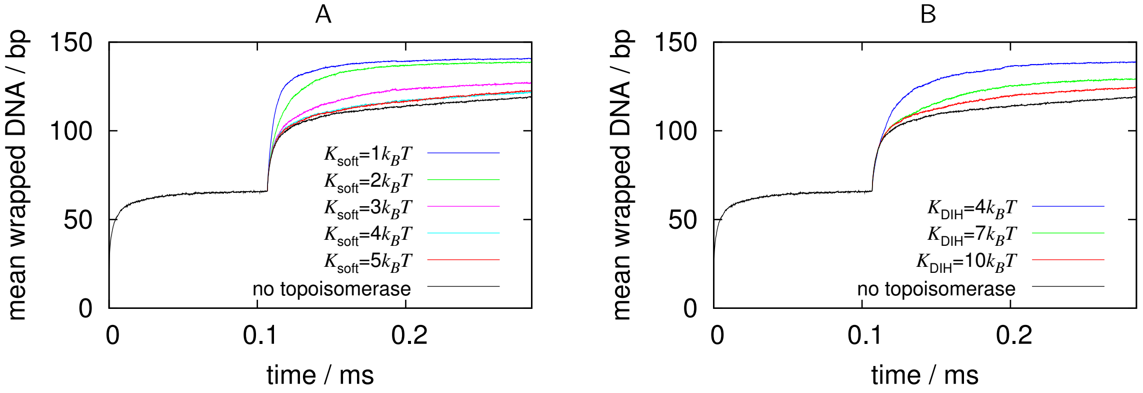

Supplementary Figure S5: Topological enzyme-like activity improves fibre quality. Plots showing how the amount of DNA wrapped around a nucleosome core, averaged over 1000 nucleosomes (100 cores in each of 10 simulations), proceeds with time during a simulation. (A) The action of topoisomerase II is introduced for 0.1 ms after the first of the two stages of the simulation; curves for different values of $K_{\rm soft}$ are shown. (B) The action of topoisomerase I is introduced for 0.1 ms after the first of the two stages of the simulation; curves for different values of $K_{\rm DIH}$ are shown. See sections II and VI of Supplementary Data. Note that these simulations were performed using the ΔLk $= -1.75$ model nucleosomes.

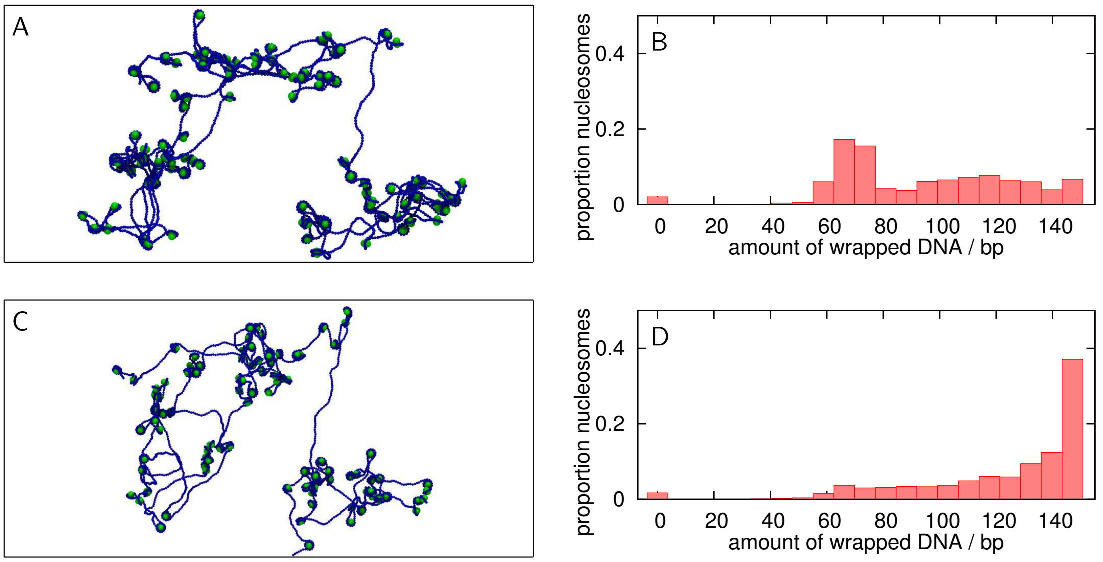

Supplementary Figure S6: Randomly positioned nucleosome cores. Initial conditions where nucleosome cores are pre-positioned at randomly chosen locations on an equilibrated DNA molecule mimic a reconstitution experiment where the DNA does not contain positioning elements in its sequence; the ΔLk $= -1.0$ model nucleosomes were used. Panels A and B show respectively a typical configuration after 0.3 ms, and a histogram of the amount of DNA wrapped for 1000 nucleosomes (100 cores in each of 10 simulations). Panels C and D show similar, but for the case where strand crossing was allowed after the first step of the reconstitution — mimicking the action of topoisomerase II.

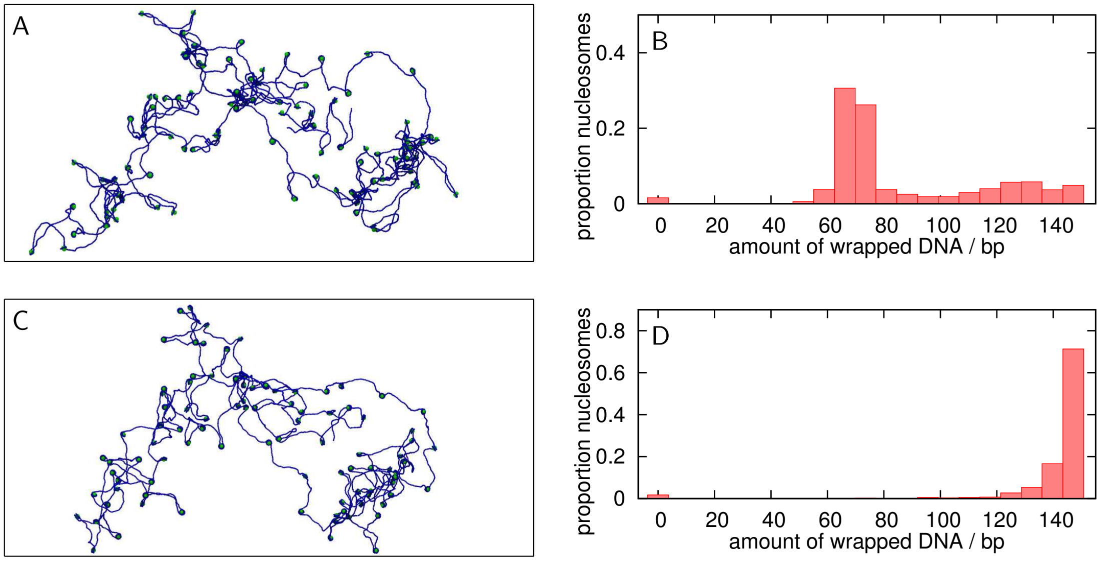

Supplementary Figure S7: Lower density nucleosome array. Using a longer DNA molecule (~ 44 kbp) with the same number of nucleosome cores results in a lower density of nucleosomes (initial condition: pre-positioned cores with regular spacing; the $\Delta\mathrm{Lk} = -1.0$ model nucleosomes were used). Panels A and B show respectively a typical configuration after 0.3 ms, and a histogram of the amount of DNA wrapped for 1000 nucleosomes (100 cores in each of 10 simulations). Panels C and D show similar, but for the case where strand crossing was allowed after the first step of the reconstitution — mimicking the action of topoisomerase II.

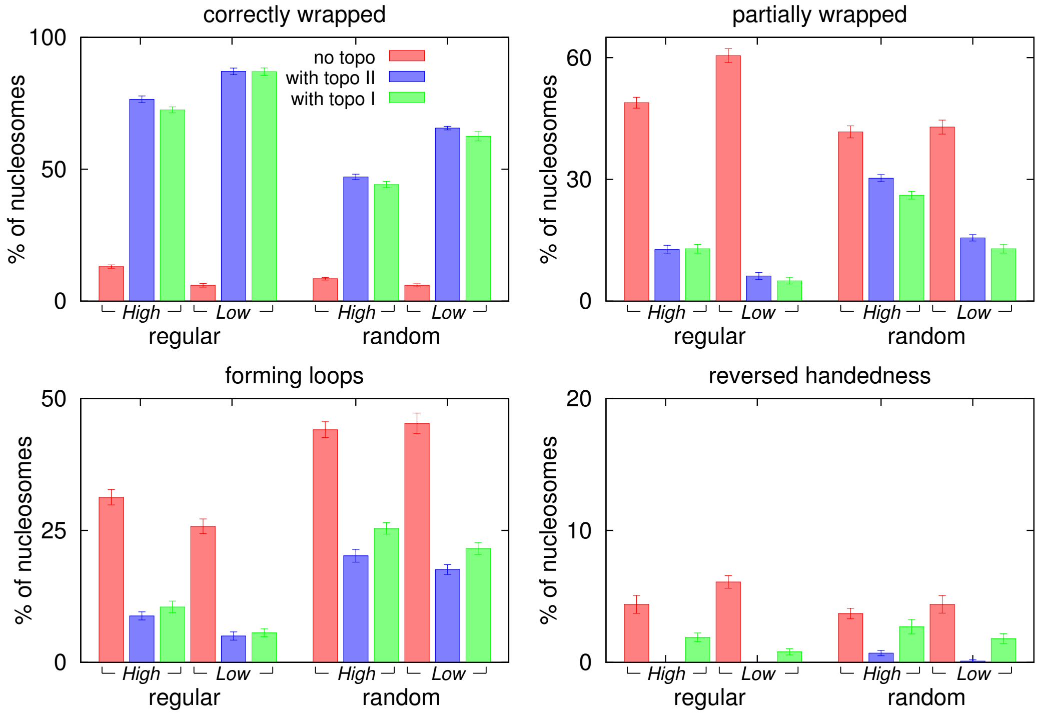

Supplementary Figure S8: Allowing type-I topoisomerase like activity also improves fibre quality. Histograms counting the numbers of correctly formed nucleosomes and defects, for three *in silico* reconstitution protocols: (i) the two-step protocol of Fig. 2 in the main text, (ii) the topo II-protocol of Fig. 4 in the main text, and (iii) a simulation where the torsional rigidity is sharply reduced following the first reconstitution step (see main text), corresponding to the activity of enzymes which can relax twist locally, such as topoisomerase I. In this figure we refer to the cases (i), (ii) and (iii) as "no topo", "topo II" and "topo I" respectively. As in Fig. 4C in the main text, we consider different initial conditions (with nucleosome cores distributed either regularly or randomly), and we analyse both high and low nucleosome density (same values as in Fig. 4C); for all cases the $\Delta\mathrm{Lk} = -1.0$ model nucleosomes were used. Each bar shows an average over 10 simulations, and error bars show the standard error of the mean. In all cases the increase in correctly formed nucleosomes on addition of topoisomerase across 10 simulations is found to be statistically significant (Welch's t-test gives $p < 10^{-5}$). For the randomly spaced nucleosomes, although the number of partially wrapped nucleosomes decreases upon addition of topoisomerase action, there is not enough evidence to confirm this change is statistically significant – this is consistent with our hypothesis that some partial wrapping defects are due to nucleosome being positioned too close together (i.e. cannot be resolved by relaxing topological constraints on the DNA). All of the other changes due to addition of topoisomerase are found to be statistically significant ($p < 10^{-2}$, Welch's t-test), except when the occurrence of the defects was anyway initially very small.

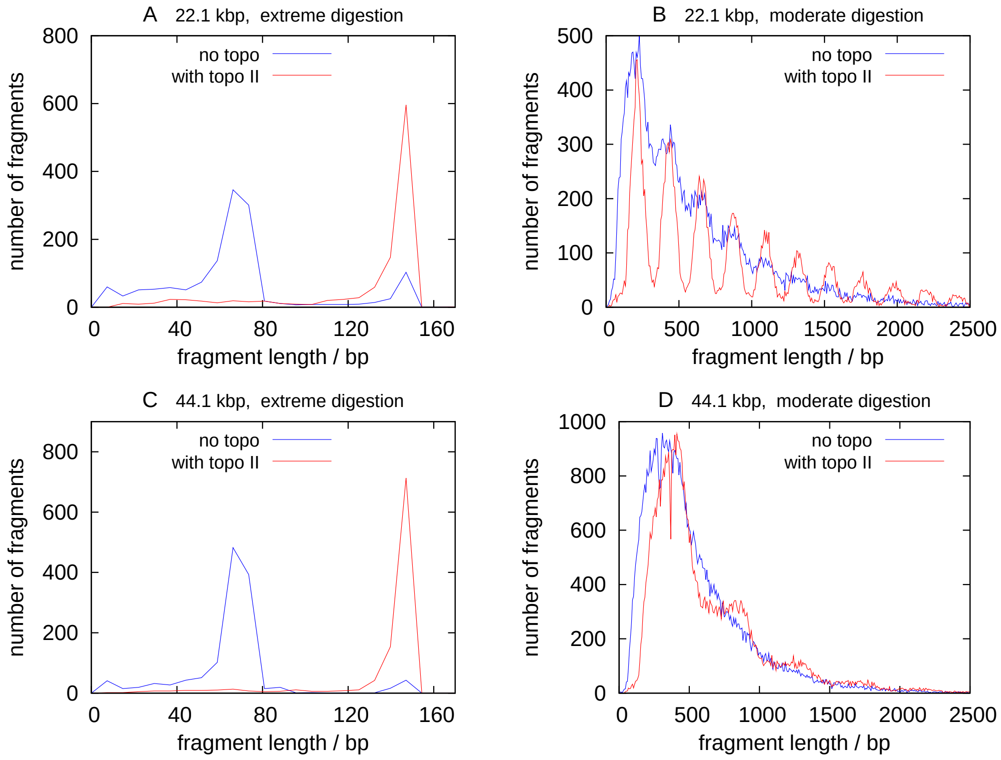

Supplementary Figure S9: Simulated nuclease digestion of *in silico* fibres leads to peaks in fragment lengths. (A) and (C) show the case of an extreme digestion, where all DNA bead not bound to nucleosomes were removed, and (B) and (D) show a moderate digestion, where the DNA was cut on average every 294 bp (see Section VII of Supplementary Data). (A) and (B) show histograms of from for the high density, 22.1 kbp DNA chromatin (data from B was used to make the simulated gel electrophoresis image in Fig. 5). (C) and (D) show similar data for the fibres with lower density regularly spaced nucleosomes (DNA length 44.1 kbp) as described in Supplementary Fig. S7. Due to the longer length of the linker DNA between nucleosome cores, the peaks in the moderate digestion plot are wider so merge together.

IX. SUPPLEMENTARY TABLE

	Regular initial positions				Random initial positions			
	Without topo-II		With topo-II		Without topo-II		With topo-II	
Density	High	Low	High	Low	High	Low	High	Low
Correctly wrapped	0.13±0.02	0.06±0.02	0.77±0.04	0.87±0.04	0.09±0.02	0.06±0.02	0.47±0.03	0.66±0.02
Partially wrapped	0.49±0.04	0.61±0.05	0.13±0.03	0.06±0.03	0.42±0.05	0.43±0.05	0.30±0.03	0.16±0.02
Forming loops	0.31±0.05	0.26±0.04	0.09±0.02	0.05±0.02	0.44±0.05	0.45±0.06	0.20±0.04	0.18±0.03
Right-handed wrapping	0.04±0.02	0.06±0.02	0±0	0±0	0.04±0.01	0.04±0.02	0.01±0.01	0.001±0.003
Not bound to DNA	0.02±0.02	0.02±0.01	0.02±0.02	0.02±0.01	0.02±0.01	0.01±0.00	0.02±0.01	0.01±0.01

Supplementary Table S1: Table showing the proportion of occurrences of different types of self-assembly defect for the various conditions. The density of nucleosomes refers to the number of nucleosomes per bp of DNA; each simulation used 100 nucleosome cores, high density simulations used 22.1 kbp DNA molecules (corresponds to a mean nucleosome separation/linker length of 74 bp — about the same as *in vivo* chromatin) and low density used 44.2 kbp DNA molecules (corresponds to a mean separation of 295 bp). Each result is an average over 10 repeat simulations, and the error shown is the standard error in the mean; in all cases the $\Delta\text{Lk} = -1.0$ model nucleosomes were used.

X. SUPPLEMENTARY MOVIE CAPTIONS

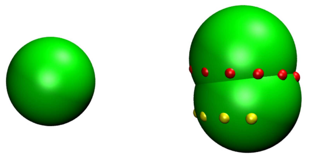

Supplementary Movie 1 Movie showing the geometry of a nanoparticle (spherical nucleosome core) and of a nucleosome core (histone octamer) with a left-handed helical binding path on its surface. For each case the movie also shows a configuration where the DNA wraps around the core. Note the right-handed helicity of the “nucleosome” for the spherical core case: for this simple model there is no way to distinguish between left-handed and right-handed nucleosomes, and this is resolved in the model considering the left-handed helical binding patch on the histone octamer surface.

Supplementary Movie 2 This movie shows the initial and final configuration of an *in silico* reconstitution experiment with nucleosome cores with a left-handed helical binding path on their surface, in the case where the simulation is started off with regularly spaced histone octamers prepositioned on the DNA (this corresponds to a sequence with strong positioning information). Close up three-dimensional details of both the initial and final configuration are shown during the movies, together with a short part of the dynamics of the self-assembled chromatin fibre in its final configuration. The movie includes final configurations for two cases: first for a two-step (see main text) reconstitution when strand crossing is disallowed, then for an analogue case in which it is permitted at the end of the first step, to mimic the action of topoisomerase II (see main text).

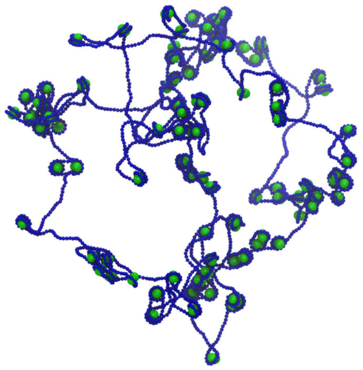

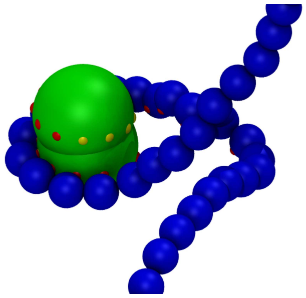

Supplementary Movie 3 This movie shows close-up views of the defects in the chromatin reconstituted *in silico* via a two-step protocol (see Fig. 2 and 3 of main text and associated discussion), which allows one to appreciate their three-dimensional structures. The defects considered are: partially wrapped nucleosomes, nucleosomes forming DNA loops, inappropriately close nucleosomes, and right-handed nucleosomes. The first, second and fourth structures share similarities with non-standard chromatin structures, respectively the hemisome or tetrasome, the dinucleosome and the reversome (see main text). The rotating view of the partially wrapped structure shows that further wrapping is inhibited by a topological entanglement which can be resolved by strand crossing.

Supplementary Movie 4 The first part of the movie shows an example of how strand crossing can resolve a partially wrapped nucleosome defect; this can be realised in practice through the action of topo-II. The second part of the movie shows how, in our simulations, the same defect can be resolved by allowing DNA to over-twist and under-twist at reduced cost, as if nicks or topo I are present. Correct wrapping now requires octamer flipping and DNA twisting close to the nucleosome; note that twist diffusion is inhibited at DNA patches when they are bound to the patches on the octamer surface.

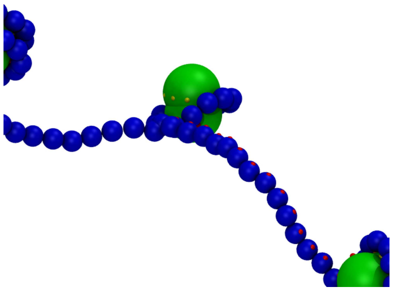

XI. REFERENCES

1. Plimpton, S. (1995) Fast Parallel Algorithms for Short-Range Molecular Dynamics. *J. Comput. Phys.*, **117**, 1–19.
2. Bishop, T. C. (2005) Molecular Dynamics Simulations of a Nucleosome and Free DNA. *J. Biomol. Struct. Dyn.*, **22**, 673–685.
3. Ettig, R., Kepper, N., Stehr, R., Wedemann, G., and Rippe, K. (2011) Dissecting DNA-Histone Interactions in the Nucleosome by Molecular Dynamics Simulations of DNA Unwrapping. *Biophysical Journal*, **101**, 1999–2008.
4. Fathizadeh, A., Berdy Besya, A., Reza Ejtehadi, M., and Schiessel, H. (2013) Rigid-body molecular dynamics of DNA inside a nucleosome. *Eur. Phys. J. E*, **36**(3), 1–10.
5. Fan, Y., Korolev, N., Lyubartsev, A. P., and Nordenski??ld, L. (2013) An Advanced Coarse-Grained Nucleosome Core Particle Model for Computer Simulations of Nucleosome-Nucleosome Interactions under Varying Ionic Conditions. *PLoS ONE*, **8**(2), e54228.
6. Chirico, G. and Langowski, J. (1994) Kinetics of DNA supercoiling studied by Brownian Dynamics Simulations. *Biopolymers*, **34**, 415–433.
7. Langowski, J. (2006) Polymer chain models of DNA and chromatin. *Eur. Phys. J. E*, **19**, 241–249.
8. Fujimoto, B. S. and Schurr, J. M. (1990) Dependence of the torsional rigidity of DNA on base composition. *Nature*, **344**, 175 – 178.
9. Zinchenko, A. A., Yoshikawa, K., and Baigl, D. (2005) Compaction of single-chain DNA by histone-inspired nanoparticles. *Phys. Rev. Lett.*, **95**, 228101.
10. Zinchenko, A. A., Sakaue, T., Araki, S., Yoshikawa, K., and Baigl, D. (2007) Single-chain compaction of long duplex DNA by cationic nanoparticles: modes of interaction and comparison with chromatin. *J. Phys. Chem. B*, **111**, 3019–3031.
11. Li, W., Dou, S.-X., and Wang, P.-Y. (2004) Brownian dynamics simulation of nucleosome formation and disruption under stretching. *J. Theor. Biol.*, **230**(3), 375 – 383.
12. Li, W., Dou, S.-X., Xie, P., and Wang, P.-Y. (2006) Brownian dynamics simulation of directional sliding of histone octamers caused by DNA bending. *Phys. Rev. E*, **73**, 051909.
13. Sakaue, T., Yoshikawa, K., Yoshimura, S. H., and Takeyasu, K. (2001) Histone Core Slips along DNA and Prefers Positioning at the Chain End. *Phys. Rev. Lett.*, **87**, 078105.
14. Dobrovolskaia, I. and Arya, G. (2012) Dynamics of Forced Nucleosome Unraveling and Role of Nonuniform Histone-DNA Interactions. *Biophys. J.*, **103**(5), 989 – 998.
15. Luger, K., Mader, A. W., Richmond, R. K., Sargent, D. F., and Richmond, T. J. (1997) Crystal structure of the nucleosome core particle at 2.8 Å resolution. *Nature*, **389**, 251–260.
16. Kulić, I. M. and Schiessel, H. (2003) Chromatin Dynamics: Nucleosomes go Mobile through Twist Defects. *Phys. Rev. Lett.*, **91**, 148103.
17. Schiessel, H. (2006) The nucleosome: A transparent, slippery, sticky and yet stable DNA-protein complex. *Eur. Phys. J. E*, **19**, 251–262.
18. Struhl, K. and Segal, E. (2013) Determinants of nucleosome positioning. *Nat. Struct. Mol. Biol.*, **20**, 267???273.
19. Anderson, J. D. and Widom, J. (2000) *J. Mol. Biol.*, **296**, 979.
20. Carey, M. F., Peterson, C. L., and Smale, S. T. Transcriptional Regulation in Eukaryotes chapter Studying Chromatin Dynamics In Vitro: Chromatin Assembly, Remodeling and Transcription Cold Spring Harbor Laboratory Press (2009).
21. Mondal, N. and Parvin, J. D. (2001) DNA topoisomerase IIα is required for RNA polymerase II transcription on chromatin templates. *Nature*, **413**, 435–438.
22. Brower-Toland, B. D., Smith, C. L., Yeh, R. C., Lis, J. T., Peterson, C. L., and Wang, M. D. (2002) Mechanical disruption of individual nucleosomes reveals a reversible multistage release of DNA. *Proceedings of the National Academy of Sciences*, **99**(4), 1960–1965.
23. Kruithof, M., Chien, F.-T., Routh, A., Logie, C., Rhodes, D., and van Noort, J. (2009) Single-molecule force spectroscopy reveals a highly compliant helical folding for the 30-nm chromatin fiber. *Nat. Struct. Mol. Biol.*, **16**, 534–540.
24. Choy, J. S. and Lee, T.-H. (2012) Structural dynamics of nucleosomes at single-molecule resolution. *Trends Biochem. Sci.*, **37**(10), 425 – 435.
25. Li, G., Levitus, M., Bustamante, C., and Widom, J. (2005) Rapid spontaneous accessibility of nucleosomal DNA. *Nat. Struct. Mol. Biol.*, **12**, 46–53.
26. Doi, M. and Edwards, S. F. (1986) The Theory of Polymer Dynamics, Oxford Science Publications, .
27. Lavelle, C. and Prunell, A. (2007) Chromatin polymorphism and the nucleosome superfamily: a genealogy. *Cell Cycle*, **6**, 2113–2119.
28. Nitiss, J. L. (2009) DNA topoisomerase II and its growing repertoire of biological functions. *Nat. Rev. Cancer*, **9**, 327–337.
29. Kornberg, R. D. and Stryer, L. (1988) Statistical distributions of nucleosomes: nonrandom locations by a stochastic mechanism. *Nucl. Acids Res.*, **16**, 6677–6689.
30. Fu, Q. Atomic Force Microscopy for Chromatin Structure Study PhD thesis Arizona State University (2010).
31. Hizume, K., Yoshimura, S. H., Maruyama, H., Joongbeak, K., and WADA, H. (2002) Chromatin reconstitution: development of a salt-dialysis method monitored by nanotechnology. *Arch. Histol. Cytol*, **65**(5), 405–413.
32. Bancaud, A., Conde e Silva, N., Barbi, M., Wagner, G., Allemand, J.-F., Mozziconacci, J., Lavelle, C., Croquette, V., Victor, J.-M., Prunell, A., and Viovy, J.-L. (2006) Structural plasticity of single chromatin fibers revealed by torsional manipulation. *Nat. Struct. Mol. Biol.*, **13**, 444.
33. Bancaud, A., Wagner, G., Conde, E. S. N., Lavelle, C., Wong, H., Mozziconacci, J., Barbi, M., Sivolob, A., Le Cam, E., Mouawad, L., Viovy, J., Victor, J., and Prunell, A. (2007) Nucleosome Chiral Transition under Positive Torsional Stress in Single Chromatin Fibers. *Mol. Cell*, **27**, 135–147.
34. Bussiek, M., Tóth, K., Brun, N., and Langowski, J. (2005) DNA-loop Formation on Nucleosomes Shown by in situ Scanning Force Microscopy of Supercoiled DNA. *J. Mol. Biol.*, **345**, 695–706.
